# Supplementary material for: Transient amplifiers of selection and reducers of fixation for death-Birth updating on graphs
Source: PLoS Comput Biol. 2020 Jan 17;16(1):e1007529. doi: 10.1371/journal.pcbi.1007529 (PMC6968840; doi:10.1371/journal.pcbi.1007529)
Supplement: S1 Appendix — This supplement contains a derivation of our weak selection method, Eqs (5)–(9), as well as analysis of our three example graph families. (PDF) [file pcbi.1007529.s001.pdf]

Transient amplifiers of selection and reducers of fixation for death-Birth updating on graphs
S1 Appendix: Mathematical derivations and proofs

Benjamin Allen Christine Sample Robert Jencks
James Withers Patricia Steinhagen Lori Brizuela
Joshua Kolodny Darren Parke Gabor Lippner
Yulia A. Dementieva

1 Model and notation

We begin with a formal description of our model as a Markov chain.

1.1 Graph

Population structure is represented by a weighted graph G with edge weights w_{ij}, $i, j \in G$. We require that G be connected, undirected ($w_{ji} = w_{ij}$ for each $i, j \in G$), and have no self-loops ($w_{ii} = 0$ for all i).

We define the *weighted degree* of vertex i as $w_i = \sum_{j \in G} w_{ij}$. The total sum of all edge weights (counting both directions for each pair of vertices) is denoted W:

$$W = \sum_{i,j \in G} w_{ij} = \sum_{i \in G} w_i.$$

The *relative weighted degree* of vertex i is defined as $\pi_i = w_i / W$.

1.2 Random walks

Our analysis makes extensive use of random walks on graphs. Steps are taken with probability proportional to edge weight. Thus the probability of a step from i to j is $p_{ij} = w_{ij}/w_i$. The probability that an n-step walk from vertex i terminates at vertex j is denoted $p_{ij}^{(n)}$.

Random walks on a weighted graph G have a stationary probability distribution, in which the probability of being at vertex i is equal to its relative weighted degree $\pi_i = w_i / \sum_j w_j$. This stationary distribution obeys the reversibility (detailed balance) property that for each $i, j \in G$,

$$\pi_i p_{ij}^{(n)} = \pi_j p_{ji}^{(n)}. \tag{1}$$

1.3 States and transitions

There are two competing types: a resident (wild) type, labeled 0, and a mutant type, labeled 1. We indicate the type currently occupying vertex $i \in G$ by the variable $x_i \in \{0,1\}$. The overall population state can be written as a binary vector $\mathbf{x} = (x_i)_{i \in G} \in \{0,1\}^G$.

Residents have fecundity (reproductive capacity) 1, while mutants have fecundity $r = 1+\delta$. Here, δ quantifies the reproductive advantage (or disadvantage, if negative) of mutants over residents. The fecundity of vertex i in a given state can be written compactly as $1+\delta x_i$. Neutral drift is represented by the case $r=1$, or equivalently, $\delta = 0$.

Under death-Birth updating, first an individual is selected, uniformly at random, to be replaced. Then a neighbor is selected, with probability proportional to fecundity times edge weight, to reproduce into the vacancy. Overall, the probability $e_{ij}(\mathbf{x})$ that vertex j is replaced by the offspring of vertex i, in state $\mathbf{x}$, can be written

$$e_{ij}(\mathbf{x}) = \frac{1}{N} \frac{w_{ij}(1+\delta x_i)}{\sum_{k \in G} w_{kj}(1+\delta x_k)}. \tag{2}$$

Offspring inherit the type of the parent.

1.4 Fixation probability

The process of resident-mutant competition is represented as a discrete-time Markov chain on $\{0,1\}^G$. This Markov chain has two absorbing states: the state $\mathbf{1}$ for which $x_i = 1$ for all $i \in G$, and the state $\mathbf{0}$ for which $x_i = 0$ for all $i \in G$. These states correspond to the fixation of mutants and residents, respectively. All other states of the evolutionary Markov chain are transient [1, Theorem 2]. Thus from any given initial state, the evolutionary Markov chain will eventually become absorbed in either state $\mathbf{0}$ or state $\mathbf{1}$.

As our initial condition, we suppose that a single mutant is placed on a vertex of G, chosen uniformly at random, and all vertices contain residents. Unless otherwise stated, we will use this initial condition throughout. We define the *fixation probability* of mutants as the probability of absorption in state $\mathbf{1}$ from this initial condition. The fixation probability of a mutant of fitness effect r on graph G is denoted $\rho_G(r)$; we will sometimes omit the G for brevity.

2 Method for weak selection

Allen et al. [2] derived a weak-selection expansion for fixation probability in terms of coalescence times. Here we provide an alternative derivation, based on results from Allen and McAvoy [3].

2.1 Change due to selection

Each vertex has a *reproductive value (RV)*, which quantifies its contribution, under neutral drift, to the future gene pool. For death-Birth updating, the reproductive value of vertex i

is $N\pi_i$ [2–5]. The RV-weighted frequency of mutants in a given state $\mathbf{x}$ is $\hat{x} = N\sum_{i\in G}\pi_i x_i$. Our method for weak selection centers on the expected change, $\Delta(\mathbf{x})$, in $\hat{x}$ from a given state $\mathbf{x}$. Note that if vertex j is replaced by the offspring of vertex i, the resulting change in $\hat{x}$ is $N\pi_j(x_i - x_j)$. Taking the expectation over all such events, with probabilities given by Eq. (2), we obtain

$$\begin{aligned}
\Delta(\mathbf{x}) &= N\sum_{i,j\in G} e_{ij}(\mathbf{x})\pi_j(x_i - x_j) \\
&= \sum_{j\in G}\pi_j\left(-x_j + \sum_{i\in G} x_i \frac{w_{ij}(1+\delta x_i)}{\sum_{k\in G} w_{kj}(1+\delta x_k)}\right) \qquad (3) \\
&= \sum_{i\in G} x_i\left(-\pi_i + \sum_{j\in G}\pi_j \frac{w_{ij}(1+\delta x_i)}{\sum_{k\in G} w_{kj}(1+\delta x_k)}\right). \qquad (4)
\end{aligned}$$

To analyze weak selection, we form a first-order Taylor expansion of $\Delta(\mathbf{x})$ in δ around $\delta = 0$. That is, we seek an expansion of the form

$$\Delta(\mathbf{x}) = \Delta^\circ(\mathbf{x}) + \delta\Delta'(\mathbf{x}) + \mathcal{O}(\delta^2). \qquad (5)$$

Above and throughout, we use a superscript $^\circ$ to indicate a quantity evaluated at neutral drift ($\delta = 0$), and a prime $'$ to indicate a derivative with respect to δ at $\delta = 0$. Expanding Eq. (4) and making use of Eq. (1), we obtain

$$\begin{aligned}
\Delta(\mathbf{x}) &= \sum_{i\in G} x_i\left(-\pi_i + \sum_{j\in G}\pi_j p_{ji}\left(1 + \delta\left(x_i - \sum_{k\in G} p_{jk}x_k\right)\right)\right) + \mathcal{O}(\delta^2) \\
&= \sum_{i\in G} x_i\left(-\pi_i + \sum_{j\in G}\pi_i p_{ij}\left(1 + \delta\left(x_i - \sum_{k\in G} p_{jk}x_k\right)\right)\right) + \mathcal{O}(\delta^2) \\
&= \delta\sum_{i\in G} x_i\left(\pi_i x_i - \pi_i\sum_{k\in G} p_{ik}^{(2)} x_k\right) + \mathcal{O}(\delta^2) \\
&= \delta\sum_{i\in G}\pi_i x_i\left(x_i - x_i^{(2)}\right) + \mathcal{O}(\delta^2).
\end{aligned}$$

Above, we have introduced the notation $x_i^{(2)} = \sum_{j\in G} p_{ij}^{(2)} x_j$. We conclude that $\Delta^\circ(\mathbf{x}) = 0$ for all states $\mathbf{x}$, and that the first-order coefficient in Eq. (5) is given by

$$\Delta'(\mathbf{x}) = \sum_{i\in G}\pi_i x_i\left(x_i - x_i^{(2)}\right). \qquad (6)$$

The appearance of $x_i^{(2)}$ reflects the fact that, when a vertex is selected for replacement, the neighbors competing to fill the vacancy are two steps from each other. Mutants co-occurring at distance two therefore affect each other's reproductive success.

2.2 Fixation probability under weak selection

We now turn to fixation probability. The neutral ($\delta = 0$) fixation probability for dB updating is $\rho^\circ = \frac{1}{N}$ [5]. We therefore seek a weak-selection expansion of the form

$$\rho(1+\delta) = \frac{1}{N} + \delta\rho' + \mathcal{O}(\delta^2). \tag{7}$$

To obtain an expression for ρ', we introduce a small rate of mutation $u > 0$ (which we will later take to zero). With each reproduction, the offspring inherits the type of the parent with probability $1-u$; otherwise, with probability u, the offspring is assigned either type 0 or 1 with equal probability. With mutation, the evolutionary Markov chain becomes ergodic [1, Theorem 1], with a unique stationary probability distribution which we call the *Mutation-Selection Stationary (MSS) distribution.* We denote expectations in this distribution by $\mathbb{E}_{\text{MSS}}$.

Allen and McAvoy [3] obtained a relationship between the expectation of $\Delta(\mathbf{x})$ under the MSS distribution, and the fixation probabilities of two competing types (with each invading the other). In the case of mutant-resident competition in the death-Birth process, Eqs. (57)-(59) of Allen and McAvoy [3] yield

$$\lim_{u\to 0} \frac{\mathbb{E}_{\text{MSS}}[\Delta]}{u} = \frac{\rho(r) - \rho(r^{-1})}{2\left(\rho(r) + \rho(r^{-1})\right)}. \tag{8}$$

We note that $\rho(r^{-1})$ is the fixation probability of the resident type into a mutant-dominated population, with the initial resident placed uniformly at random. This is because a resident of fitness 1 invading a mutant population of fitness r is equivalent (upon rescaling by r) to a mutant of fitness r^{-1} invading a resident population of fitness 1.

For neutral drift ($r = 1$), we have $\Delta^\circ(\mathbf{x}) = 0$ for all states $\mathbf{x}$, and $\rho^\circ = \frac{1}{N}$ [5]. Taking the derivative of both sides of Eq. (8) at $r = 1$ yields

$$\lim_{u\to 0} \frac{\mathbb{E}^\circ_{\text{MSS}}[\Delta']}{u} = \frac{\rho'}{2\rho^\circ} = \frac{N\rho'}{2}. \tag{9}$$

The notation $\mathbb{E}^\circ_{\text{MSS}}$ means that the expectation is taken over the *neutral* ($\delta = 0$) MSS distribution. Combining Eqs. (6) and (9), we have

$$\rho' = \frac{2}{N} \lim_{u\to 0} \frac{\mathbb{E}^\circ_{\text{MSS}}[\Delta']}{u} = \frac{2}{N} \sum_{i\in G} \pi_i \lim_{u\to 0} \left(\frac{\mathbb{E}^\circ_{\text{MSS}}\left[x_i\left(x_i - x_i^{(2)}\right)\right]}{u} \right). \tag{10}$$

2.3 Reduction to coalescence times

Eq. (10) expresses ρ' in terms of a particular statistic of spatial assortment under the MSS distribution. Such statistics can be calculated in terms of the coalescence times τ_{ij}, which are the unique solution to

$$\tau_{ij} = \begin{cases} 0 & i = j \\ 1 + \frac{1}{2}\sum_{k\in G}\left(p_{ik}\tau_{jk} + p_{jk}\tau_{ik}\right) & i \neq j. \end{cases} \tag{11}$$

Intuitively, the larger the coalescence time τ_{ij}, the more the occupants of i and j are separated from their common ancestor, and the less likely they are to have the same type. Specifically, Eq. (111) of Allen and McAvoy [3] gives the relationship

$$\lim_{u\to 0} \frac{\mathbb{E}^{\circ}_{\mathrm{MSS}}[x_i(x_i - x_j)]}{u} = \frac{\tau_{ij}}{4}. \tag{12}$$

We define $\tau^{(n)}$ as the expected coalescence time from the two ends of an n-step stationary random walk:

$$\tau^{(n)} = \sum_{i.j\in G} \pi_i p_{ij}^{(n)} \tau_{ij}. \tag{13}$$

Multiplying Eq. (12) by $\pi_i p_{ij}^{(2)}$ and summing over all $i, j \in G$, we obtain

$$\sum_{i\in G} \pi_i \lim_{u\to 0} \frac{\mathbb{E}^{\circ}_{\mathrm{MSS}}[x_i(x_i - x_i^{(2)})]}{u} = \frac{\tau^{(2)}}{4}. \tag{14}$$

where we recall the notation $x_i^{(2)} = \sum_{j\in G} p_{ij}^{(2)} x_j$. Combining Eqs. (10) and (14) yields

$$\rho' = \frac{\tau^{(2)}}{2N}. \tag{15}$$

We observe that $\tau^{(2)}$ characterizes the frequency with which mutants co-occur (and therefore compete with each other) at distance two.

Allen et al. [2] derived the following recurrence relation for $\tau^{(n)}$:

$$\tau^{(n+1)} = \tau^{(n)} + \sum_{i\in G} \pi_i p_{ii}^{(n)} \tau_i - 1. \tag{16}$$

Above, τ_i is the remeeting time from vertex i:

$$\tau_i = 1 + \sum_{j\in G} p_{ij} \tau_{ij}. \tag{17}$$

Noting that $p_{ii}^{(0)} = 1$ and $p_{ii}^{(1)} = 0$ (since G has no self-loops), we have

$$\tau^{(0)} = 0 \tag{18}$$

$$\tau^{(1)} = \sum_{i\in G} \pi_i \tau_i - 1 \tag{19}$$

$$\tau^{(2)} = \sum_{i\in G} \pi_i \tau_i - 2. \tag{20}$$

As in the main text, we define effective population size as

$$N_{\mathrm{eff}} = \sum_{i\in G} \pi_i \tau_i. \tag{21}$$

Combining Eqs. (7), (15), (20), and (21) we obtain the expansion

$$\rho(1+\delta) = \frac{1}{N} + \delta\frac{N_{\text{eff}}-2}{2N} + \mathcal{O}(\delta^2), \tag{22}$$

which is Eq. (8) of the main text.

Thus a weak-selection expansion of fixation probabilities can be obtained for any graph by first computing pairwise coalescence times, and using these to compute N_{eff}. This method can be performed in $\mathcal{O}(N^6)$ time using standard methods such as Gaussian elimination.

3 Perturbation of isothermal graphs

Here we derive Eq. (13) of the main text, which provides a recipe for perturbing an isothermal graph into an amplifier of weak selection.

Consider a family of graphs indexed by ϵ, which is isothermal when $\epsilon = 0$. We will use a superscript $^{(0)}$ to indicate a quantity evaluated at $\epsilon = 0$, and a superscript $^{(1)}$ to indicate an ϵ-derivative taken at $\epsilon = 0$.

Since the graph is isothermal when $\epsilon = 0$, we have $\pi_i^{(0)} = 1/N$ for all $i \in G$. Taking the ϵ-derivative of the identity $\sum_{i\in G} \pi_i^2 \tau_i = 1$ at $\epsilon = 0$ yields

$$\begin{aligned}
0 &= \left(\sum_{i\in G} \pi_i^2 \tau_i\right)^{(1)} \\
&= 2\sum_{i\in G} \pi_i^{(0)}\pi_i^{(1)}\tau_i^{(0)} + \sum_{i\in G}\left(\pi_i^{(0)}\right)^2 \tau_i^{(1)} \\
&= \frac{2}{N}\sum_{i\in G} \pi_i^{(1)}\tau_i^{(0)} + \frac{1}{N^2}\sum_{i\in G}\tau_i^{(1)}.
\end{aligned}$$

This implies

$$\frac{1}{N}\sum_{i\in G}\tau_i^{(1)} = -2\sum_{i\in G}\pi_i^{(1)}\tau_i^{(0)}. \tag{23}$$

Let us now consider how the perturbation affects the effective population size, N_{eff}. Since the graph is isothermal when $\epsilon = 0$, we have $N_{\text{eff}}^{(0)} = N$. Therefore, if $N_{\text{eff}}^{(1)}$ is positive, we will have $N_{\text{eff}} > N$ for all sufficiently small $\epsilon > 0$, meaning we will have created a family of amplifiers of weak selection.

Differentiating Eq (21) at $\epsilon = 0$ gives

$$\begin{aligned}
N_{\text{eff}}^{(1)} &= \sum_{i\in G}\pi_i^{(1)}\tau_i^{(0)} + \sum_{i\in G}\pi_i^{(0)}\tau_i^{(1)} \\
&= \sum_{i\in G}\pi_i^{(1)}\tau_i^{(0)} + \frac{1}{N}\sum_{i\in G}\tau_i^{(1)}.
\end{aligned}$$

Substituting from Eq. (23), we have

$$N_{\text{eff}}^{(1)} = \sum_{i\in G} \pi_i^{(1)}\tau_i^{(0)} - 2\sum_{i\in G} \pi_i^{(1)}\tau_i^{(0)}$$
$$= -\sum_{i\in G} \pi_i^{(1)}\tau_i^{(0)}. \tag{24}$$

This is Eq. (13) of the main text. It follows that to construct an amplifier of weak selection, one must perturb in such a way that $\sum_{i\in G} \pi_i^{(1)}\tau_i^{(0)} < 0$, which involves decreasing the weighted degree of vertices with large remeeting time and/or increasing the weighted degree of vertices with small remeeting time.

4 Mathematical lemmas

Here we prove mathematical lemmas that will be used to characterize the behavior of our example families of graphs in Section 5. In analyzing these examples, we repeatedly encounter the function

$$F_r(x) = \ln\left|\frac{1-r^{-x}}{x}\right|, \tag{25}$$

defined for $x > 0, r > 0, r \neq 1$. Before proceeding, we establish some results about this function and about convex functions in general.

Lemma 1. *$F_r(x)$ is increasing in x for $0 < r < 1$, decreasing in x for $r > 1$, and convex in x for all $r > 0, r \neq 1$.*

Proof. To prove the monotonicity of $F_r(x)$, we examine the first derivative:

$$F_r'(x) = \frac{\ln(r^x)}{x(r^x-1)} - \frac{1}{x}.$$

We note that for all $y > 0,\ y \neq 1$,

$$\ln(y) < y - 1. \tag{26}$$

In particular,

$$\ln(r^x) < r^x - 1. \tag{27}$$

In the case $r > 1$ (recalling that $x > 0$) we have $x(r^x - 1) > 0$. Dividing inequality (27) by $x(r^x - 1)$ we obtain

$$\frac{\ln(r^x)}{x(r^x-1)} > \frac{1}{x},$$

which shows that $F_r'(x) > 0$. In the case $0 < r < 1$ we have $x(r^x - 1) < 0$ and therefore

$$\frac{\ln(r^x)}{x(r^x-1)} < \frac{1}{x},$$

which shows that $F_r'(x) < 0$. This proves that $F_r(x)$ is increasing in x for $r > 1$ and decreasing for $0 < r < 1$.

To prove the convexity of $F_r(x)$, we examine the second derivative,

$$F_r''(x) = \frac{\left(r^x - 1\right)^2 - r^x \ln^2\left(r^x\right)}{x^2(r^x - 1)^2}. \tag{28}$$

The denominator is positive for all valid r and x, so we must show the numerator is positive as well. We rearrange inequality Eq. (26) to

$$2y > 2(1 + \ln y).$$

For $r > 1$, integrating both sides from $y = 1$ to $y = r^{x/2}$ gives

$$r^x - 1 > r^{x/2} \ln\left(r^x\right)$$

Squaring both sides gives

$$(r^x - 1)^2 > r^x \ln^2\left(r^x\right). \tag{29}$$

A similar argument obtains the same result for the case $0 < r < 1$. Thus the numerator of $F_r''(x)$ in Eq. (28) is positive. This proves that $F_r(x)$ is strictly convex in x for all $r > 0, r \neq 1$. □

Lemma 2. *Let $f(x)$ be a twice-differentiable, strictly convex function defined on some interval $[a, b]$. Then for any d such that $0 < d < (b - a)/2$,*

$$f(a + d) + f(b - d) < f(a) + f(b). \tag{30}$$

Proof. By the Mean Value Theorem, there exists $c_1 \in (a, a + d)$ such that

$$f'(c_1) = \frac{f(a + d) - f(a)}{d}. \tag{31}$$

Similarly, there exists $c_2 \in (b - d, b)$ such that

$$f'(c_2) = \frac{f(b) - f(b - d)}{d}. \tag{32}$$

Because $d < (b-a)/2$, $c_2 > c_1$. Since f is strictly convex, its derivative is strictly increasing. Thus, $f'(c_2) > f'(c_1)$. Combining with Eqs. (31) and (32) yields

$$f(b) - f(b - d) > f(a + d) - f(a).$$

Rearranging yields the desired result. □

5 Examples

We consider three examples: the m-Fan, Star of Islands, and Star of Islands with Separated Hubs. These graphs act conditionally as reducers, suppressors, or transient amplifiers.

5.1 Separated Hubs

The Separated Hubs graph has $h \geq 1$ "hub" vertices, $n \geq 2$ "blades", and $m \geq 2$ vertices per blade. Within each blade, vertices are connected to one another with weight 1. Each hub vertex is also connected to each blade vertex with weight ϵ. The Fan graph is the $h = 1$ case of Separated Hubs.

5.1.1 Weak Selection

Let H represent a hub vertex and B represent any blade vertex. The weighted degree and relative weighted degrees of these vertices are:

$$\begin{aligned} w_H &= nm\epsilon \\ w_B &= m - 1 + h\epsilon \\ \pi_H &= \frac{w_H}{hw_H + nmw_B} = \frac{\epsilon}{2h\epsilon + m - 1} \\ \pi_B &= \frac{w_B}{hw_H + nmw_B} = \frac{h\epsilon + m - 1}{nm(2h\epsilon + m - 1)}. \end{aligned}$$

Let B and B' represent any two vertices on the same blade and H and H' represent any two hub vertices. The step probabilities are:

$$p_{BB'} = \frac{1}{w_B} = \frac{1}{m - 1 + h\epsilon} \tag{33}$$

$$p_{BH} = \frac{\epsilon}{w_B} = \frac{\epsilon}{m - 1 + h\epsilon} \tag{34}$$

$$p_{HB} = \frac{\epsilon}{w_H} = \frac{1}{nm}. \tag{35}$$

All other step probabilities (e.g. between vertices on different blades) are zero.

When solving Eq. (11), there are four coalescence times to consider: $\tau_{HH'}$ for two different hub vertices, τ_{HB} for a hub and a blade, $\tau_{BB'}$ for two different vertices on a common blade, and $\tau_{BB''}$ for two vertices on different blades. Note that $\tau_{ij} = \tau_{ji}$.

For $\tau_{HH'}$, a walker from a hub vertex can step to any of the nm blade vertices, with probability p_{HB} each, and the resulting coalescence time is τ_{HB}. Since the two walkers are interchangeable in this case, the factor of 1/2 is eliminated, resulting in

$$\tau_{HH'} = 1 + nmp_{HB}\tau_{HB}. \tag{36}$$

For τ_{HB}, we have the following possibilities:

- Blade walker steps to one of $h - 1$ other hub vertices, with probability p_{BH} each, and resulting coalescence time $\tau_{HH'}$.
- Blade walker vertex steps to one of the other $m - 1$ vertices on the same blade, with probability $p_{BB'}$ each, and resulting coalescence time τ_{HB}.

- Hub walker steps to one of $m-1$ vertices on the same blade as the other walker, with probability p_{HB} each, and resulting coalescence time $\tau_{BB'}$.
- Hub walker steps to on of the $(n-1)m$ vertices on other blades, with probability p_{HB} each and resulting coalescence time $\tau_{BB''}$.

(Above and throughout, we exclude steps that bring one walker to the same vertex as the other, because the resulting coalescence time is zero.) Summing these possibilities gives

$$\tau_{HB} = 1 + \frac{1}{2}\big((h-1)p_{BH}\tau_{HH'} + (m-1)p_{BB'}\tau_{HB} + (m-1)p_{HB}\tau_{BB'} + (n-1)mp_{HB}\tau_{BB''}\big). \tag{37}$$

For $\tau_{BB'}$, a walker from a blade vertex can step to one of the remaining $m-2$ vertices on its blade, with probability $p_{BB'}$ each and resulting coalescence time $\tau_{BB'}$. Alternatively, the walker can step to one of the h hub vertices with probability p_{BH} and resulting coalescence time τ_{HB}. This gives

$$\tau_{BB'} = 1 + (m-2)p_{BB'}\tau_{BB'} + hp_{BH}\tau_{HB}. \tag{38}$$

For $\tau_{BB''}$, a walker can either step to one of the $m-1$ vertices on its own blade (probability $p_{BB'}$ each and resulting coalescence time $\tau_{BB'}$) or to one of the h hub vertices (probability p_{BH} each and resulting coalescence time τ_{HB}).

$$\tau_{BB''} = 1 + (m-1)p_{BB'}\tau_{BB''} + 2hp_{BH}\tau_{HB}. \tag{39}$$

Solving the system, Eqs. (36)–(39), and substituting into Eqs. (17) and (21), we obtain

$$N_{\text{eff}} = N + \frac{\text{num}}{\text{denom}} \tag{40}$$

where

$$\begin{aligned} \text{num} &= \big(m-1-\epsilon(nm-h)\big) \\ &\times \big(m(m-1)(n-h-1) + \epsilon h(nm^2+nm-3mh-m+h+1) + 2\epsilon^2 h^2(nm-h)\big), \end{aligned} \tag{41}$$

$$\text{denom} = (m-1+2\epsilon h)\big(m(m-1) + \epsilon(nm+2mh-h) + \epsilon^2 h(nm+h)\big). \tag{42}$$

For $\epsilon = (m-1)/(nm-h)$, the graph is isothermal and we have $N_{\text{eff}} = N$, as expected. For $\epsilon \to 0$, the effective population size becomes

$$\lim_{\epsilon\to 0} N_{\text{eff}} = nm + n - 1. \tag{43}$$

Since the population size is $N = nm + h$, we find that the Separated Hubs graph with $\epsilon \to 0$ is an amplifier of weak selection for $n > h+1$ and a suppressor of weak selection for $n < h+1$. We show below that these results extend to nonweak selection as well.

From Eq. (22), we have the first-order term of the weak-selection fixation probability:

$$\lim_{\epsilon\to 0} \rho' = \frac{nm+n-3}{2(h+nm)}. \tag{44}$$

Since the right-hand side is decreasing in h, the optimal amplifier of weak selection occurs when $h = 1$, which is the case of the Fan (Section 5.2).

5.1.2 Nonweak Selection

We now calculate fixation probabilities, for arbitrary mutant fitness r, on the Separated Hubs graph in the $\epsilon \to 0$ limit. This limit leads to a separation of timescales: a fast timescale for events whose probability is $\mathcal{O}(1)$ as $\epsilon \to 0$, and a slow timescale for events with probability $\mathcal{O}(\epsilon)$. Fixation on each blade vertex occurs on the fast timescale. Replacement of hub vertices by individuals on the blades also occurs on the fast timescale. Changes in the number of blade vertices that are fixed for mutants occurs on the slow timescale.

Since replacement of hub vertices occurs on the fast timescale, we assume that the types of the hubs converge in time-average to their stationary probability distribution. To determine this distribution, we note that, when a hub is chosen for replacement, the probability to be replaced by a mutant is proportional to rkm, while the probability to be replaced by a resident is proportional to $n-k$, where k denotes the number of blades that are fixed for mutants. Therefore, for each hub vertex, the stationary probabilities are given by

$$\mathbb{P}[\text{Hub is M}] = \frac{rk}{rk+n-k}$$
$$\mathbb{P}[\text{Hub is R}] = \frac{n-k}{rk+n-k}.$$

The types of different hub vertices are independent in the stationary distribution.

Dynamics on the slow timescale can be represented as a continuous-time Markov chain. We identify the states according to the number $k \in \{0, \ldots, n\}$ of blades that are fixed for mutants (with the remaining $n-k$ fixed for residents). Let us derive the transition rate from state k to state $k+1$. First, a resident blade vertex must be chosen for replacement, which happens with probability

$$\mathbb{P}[\text{Blade R dies}] = \frac{m(n-k)}{nm+h}. \tag{45}$$

Then a mutant from the hub must be chosen to replace this resident blade vertex. The probability of this is

$$\mathbb{P}[\text{Hub M replaces}] = \mathbb{E}\left[\frac{\epsilon r X}{\epsilon(rX+h-X)+m-1}\right].$$

Above, X is a random variable representing the number of mutant hubs in this state, with distribution

$$X \sim \text{Binom}\left(h, \frac{rk}{rk+n-k}\right).$$

As $\epsilon \to 0$, the probability of a hub mutant replacing the vacancy becomes

$$\mathbb{P}[\text{Hub M replaces}] = \frac{\epsilon r^2 hk}{(rk+n-k)(m-1)} + \mathcal{O}(\epsilon^2). \tag{46}$$

Finally, the mutant type must become fixed on the blade. Since each blade is a complete graph of size m, this happens with probability

$$\mathbb{P}[\text{M fixes on blade}] = \frac{m-1}{m}\frac{1-r^{-1}}{1-r^{-(m-1)}}. \tag{47}$$

Combining Eqs. (45), (46), and (47) and neglecting terms of order ϵ^2, the transition rate from state k to $k+1$ for the slow timescale is

$$Q_{k,k+1} = \left(\frac{m(n-k)}{nm+h}\right)\left(\frac{\epsilon r^2 hk}{(rk+n-k)(m-1)}\right)\left(\frac{m-1}{m}\frac{1-r^{-1}}{1-r^{-(m-1)}}\right). \tag{48}$$

A similar argument yields the transition rate from state k to $k-1$:

$$Q_{k,k-1} = \left(\frac{mk}{nm+h}\right)\left(\frac{\epsilon h(n-k)}{(rk+n-k)r(m-1)}\right)\left(\frac{m-1}{m}\frac{1-r}{1-r^{m-1}}\right). \tag{49}$$

We observe that

$$\frac{Q_{k,k-1}}{Q_{k,k+1}} = r^{-(m+1)}, \tag{50}$$

independently of k.

The probability of fixation starting with one blade is:

$$\begin{aligned}\lim_{t\to\infty} Q_{1,n}^{(t)} &= \frac{1}{1+\sum_{k=1}^{n-1}\prod_{j=1}^{k}\frac{Q_{j,j-1}}{Q_{j,j+1}}}\\ &= \frac{1}{1+\sum_{k=1}^{n-1} r^{-k(m+1)}}\\ &= \frac{1-r^{-(m+1)}}{1-r^{-n(m+1)}}.\end{aligned}$$

In the $\epsilon \to 0$ limit, fixation becomes impossible for mutants originating on a hub vertex, since these vertices are much more likely to be replaced than to be chosen for reproduction. Thus the overall fixation probability is equal to the probability that a mutant is placed on a blade, multiplied by the probability that it fixates on the blade, multiplied by the probability of fixation from one blade:

$$\begin{aligned}\rho_{SH_{n,m,h}}(r) &= \left(\frac{nm}{nm+h}\right)\left(\frac{m-1}{m}\frac{1-r^{-1}}{1-r^{-(m-1)}}\right)\left(\frac{1-r^{-(m+1)}}{1-r^{-n(m+1)}}\right)\\ &= \left(\frac{n(m-1)}{nm+h}\right)\left(\frac{1-r^{-1}}{1-r^{-(m-1)}}\right)\left(\frac{1-r^{-(m+1)}}{1-r^{-n(m+1)}}\right).\end{aligned} \tag{51}$$

In the limit of many blades, we obtain

$$\lim_{n\to\infty}\rho_{SH_{n,m,h}}(r) = \begin{cases} 0 & 0 \le r \le 1\\ \frac{(m-1)(1-r^{-1})(1-r^{-(m+1)})}{m(1-r^{-(m-1)})} & r > 1.\end{cases} \tag{52}$$

Interestingly, this limit is independent of h, the number of hubs.

We are now prepared to prove a complete classification of the behavior of the Separated Hubs graph in the $\epsilon \to 0$ limit:

Theorem 3. *The Separated Hubs graph $SH_{n,m,h}$ is, in the $\epsilon \to 0$ limit,*

- *a suppressor for $n \leq h$,*
- *a reducer for $n = h + 1$,*
- *a transient amplifier for $n \geq h + 2$.*

Proof. We wish to compare the fixation probability on the Separated Hubs graph to that of a complete graph of equal size $N = nm + h$. The ratio of fixation probabilities is

$$\frac{\rho_{SH_{n,m,h}}(r)}{\rho_{K_{nm+h}}(r)} = \frac{\left(\dfrac{1 - r^{-(m+1)}}{m+1}\right)\left(\dfrac{1 - r^{-(nm+h-1)}}{nm+h-1}\right)}{\left(\dfrac{1 - r^{-(m-1)}}{m-1}\right)\left(\dfrac{1 - r^{-n(m+1)}}{n(m+1)}\right)}. \tag{53}$$

The logarithm of this ratio can be written in terms of the function $F_r(x) = \ln \left| \frac{1-r^{-x}}{x} \right|$:

$$\ln\left(\frac{\rho_{SH_{n,m,h}}(r)}{\rho_{K_{nm+h}}(r)}\right) = F_r(m+1) + F_r(nm+h-1) - F_r(m-1) - F_r(nm+n). \tag{54}$$

We prove the cases in increasing order of difficulty.

Case 1, $n = h + 1$: Substituting $h = n - 1$ into Eq. (54) gives

$$\ln\left(\frac{\rho_{SH_{n,m,n-1}}(r)}{\rho_{K_{nm+n-1}}(r)}\right) = F_r(m+1) + F_r(nm+n-2) - F_r(m-1) - F_r(nm+n).$$

By Lemma 1 $F_r(x)$ is convex in x for all $r \neq 1$. Applying Lemma 2 with $a = m - 1$, $b = nm + n$, and $d = 2$, we obtain

$$\ln\left(\frac{\rho_{SH_{n,m,n-1}}(r)}{\rho_{K_{nm+n-1}}(r)}\right) = F_r(m+1) + F_r(nm+n-2) - F_r(m-1) - F_r(nm+n) < 0. \tag{55}$$

Therefore $\rho_{SH_{n,m,n-1}}(r) < \rho_{K_{nm+n-1}}(r)$ for all $r \neq 1$, proving $SH_{n,m,h}$ is a reducer in this case.

Case 2, $n \leq h$: Here we wish to prove that $SH_{n,m,h}$ is a suppressor. We break into three subcases.

Subcase 2.1, $r > 1$: By Lemma 1, $F_r(x)$ is decreasing in x for $r > 1$. Therefore, for $n \leq h$ and $r > 1$,

$$F_r(nm+h-1) < F_r(nm+n-2). \tag{56}$$

Combining with the inequality in Eq. (55), we have

$$\ln\left(\frac{\rho_{SH_{n,m,h}}(r)}{\rho_{K_{nm+h}}(r)}\right) = F_r(m+1) + F_r(nm+h-1) - F_r(m-1) - F_r(nm+n) < 0.$$

Thus $\rho_{SH_{n,m,h}}(r) < \rho_{K_{nm+h}}(r)$ for $n \leq h$ and $r > 1$.

Subcase 2.2, $0 < r < 1$ *and* $n \leq h - 1$: By Lemma 1, $F_r(x)$ is increasing in x for $0 < r < 1$. Therefore, in this subcase, we have $F_r(m+1) > F_r(m-1)$ and $F_r(nm+h-1) \geq F_r(nm+n)$. Combining these two inequalities gives

$$\ln\left(\frac{\rho_{SH_{n,m,h}}(r)}{\rho_{K_{nm+h}}(r)}\right) = F_r(m+1) + F_r(nm+h-1) - F_r(m-1) - F_r(nm+n) > 0. \quad (57)$$

Thus $\rho_{SH_{n,m,h}}(r) > \rho_{K_{nm+h}}(r)$ for $0 < r < 1$ and $n \leq h - 1$.

Subcase 2.3, $0 < r < 1$ *and* $n = h$: Substituting $h = n$ into Eq. (54) gives

$$\ln\left(\frac{\rho_{SH_{n,m,n}}(r)}{\rho_{K_{nm+n}}(r)}\right) = F_r(m+1) - F_r(m-1) - \left(F_r(nm+n) - F_r(nm+n-1)\right). \quad (58)$$

First consider the difference $F_r(m+1) - F_r(m-1)$. By Lemma 1, $F_r(x)$ is convex and increasing when $0 < r < 1$. Thus, $F_r(m+1) - F_r(m-1)$ is minimized when m is smallest ($m = 2$):

$$F_r(m+1) - F_r(m-1) \geq F_r(3) - F_r(1). \quad (59)$$

Next consider the difference $F_r(nm+n) - F_r(nm+n-1)$. By the Mean Value Theorem, there is some $c \in (nm+n-1, nm+n)$ such that

$$F_r(nm+n) - F_r(nm+n-1) = F_r'(c). \quad (60)$$

Since $F_r'(x)$ is increasing in x for $0 < r < 1$ by Lemma 1, we have

$$F_r'(c) < \lim_{x\to\infty} F_r'(x) = \lim_{x\to\infty}\left(\frac{\ln(r^x)}{x(r^x - 1)} - \frac{1}{x}\right) = -\ln(r).$$

Combining with Eq. (60) gives

$$F_r(nm+n) - F_r(nm+n-1) < -\ln(r). \quad (61)$$

Combining Eqs. (58), (59), and (61) gives the inequality

$$\ln\left(\frac{\rho_{SH_{n,m,h}}(r)}{\rho_{K_{nm+h}}(r)}\right) > F_r(3) - F_r(1) + \ln r.$$

The right-hand side above is equal to $\ln\left(1 + \frac{(r-1)^2}{3r}\right)$, which is positive for $0 < r < 1$. Therefore $\rho_{SH_{n,m,h}}(r) > \rho_{K_{nm+h}}(r)$ for $0 < r < 1$ and $n = h$.

Combining the three subcases of Case 2, we have shown that when $n \leq h$, we have $\rho_{SH_{n,m,h}}(r) > \rho_{K_{nm+h}}(r)$ for $0 < r < 1$ and $\rho_{SH_{n,m,h}}(r) < \rho_{K_{nm+h}}(r)$ for $r > 1$. This proves that the Separated Hubs graph is a suppressor for $n \leq h$.

Case 3, $n \geq h+2$: Here we wish to prove that $SH_{n,m,h}$ is a transient amplifier. We break into three subcases.

Subcase 3.1, $0 < r < 1$: By Lemma 1, $F_r(x)$ is increasing in x for $0 < r < 1$. Since $h \leq n-2$, we have $F_r(nm+h-1) < F_r(nm+n-2)$. Combining with Eqs. (54) and (55) gives

$$\ln\left(\frac{\rho_{SH_{n,m,h}}(r)}{\rho_{K_{nm+h}}(r)}\right) = F_r(m+1) + F_r(nm+h-1) - F_r(m-1) - F_r(nm+n) < 0. \quad (62)$$

This proves that $\rho_{SH_{n,m,h}}(r) < \rho_{K_{nm+h}}(r)$ for $n \geq h+2$ and $0 < r < 1$.

Subcase 3.2, $r > 1$: To complete the proof that the Separated Hubs graph is a transient amplifier for $n \geq h+2$, we must show that $\rho_{SH_{n,m,h}}(r) > \rho_{K_{nm+h}}(r)$ only on some connected interval $1 < r < r^*$. We already know that $\rho_{SH_{n,m,h}}(1) = \rho_{K_{nm+h}}(1) = 1/N$, and that $\rho_{SH_{n,m,h}}$ is an amplifier of weak selection, meaning that $\rho_{SH_{n,m,h}}(r) > \rho_{K_{nm+h}}(r)$ for some interval $1 < r < r^*$. It remains only to prove that the set of r-values for which $\rho_{SH_{n,m,h}}(r) > \rho_{K_{nm+h}}(r)$ is connected. This amounts to showing that the equation $\rho_{SH_{n,m,h}}(r) = \rho_{K_{nm+h}}(r)$ has only one solution with $r > 1$.

To show this, we set the ratio of fixation probabilities equal to one:

$$1 = \frac{\rho_{SH_{n,m,h}}(r)}{\rho_{K_{nm+h}}(r)} = \frac{\left(\dfrac{1-r^{-(m+1)}}{m+1}\right)\left(\dfrac{1-r^{-(nm+h-1)}}{nm+h-1}\right)}{\left(\dfrac{1-r^{-(m-1)}}{m-1}\right)\left(\dfrac{1-r^{-n(m+1)}}{n(m+1)}\right)}. \quad (63)$$

This is equivalent to

$$(nm+h-1)\left(r^{m-1}-1\right)\left(r^{nm+n}-1\right) - n(m-1)\left(r^{m+1}-1\right)\left(r^{nm+h-1}-1\right)r^{n-h-1} = 0. \quad (64)$$

The left-hand side above expands to the polynomial

$$(n+h-1)r^{nm+m+n-1} - (nm+h-1)r^{nm+n} + n(m-1)r^{nm+n-2} + n(m-1)r^{n+m-h} - n(m-1)r^{n-h-1} - (nm+h-1)r^{m-1} + nm+h-1. \quad (65)$$

We will use Descartes' rule of signs [6] to show that this polynomial has exactly one root greater than 1. Given $m \geq 2$ and $n-2 \geq h \geq 1$, the terms of the polynomial of Eq. (65) are in descending order of exponent, with the possible exception of the fifth and sixth terms. Since the quantities $n+h-1$, $nm+h-1$, and $n(m-1)$ are all positive, there are four sign changes between the consecutive coefficients of this polynomial. (The coefficients of the fifth and sixth terms are both negative so their ordering does not matter.) By Descartes' rule of signs, this polynomial can have zero, two, or four positive roots, counting multiplicity. From Eq. (64) we infer that $r = 1$ is at least a double root of this polynomial, since both terms of the left-hand side Eq. (64) contain a factor of the form $(r^x - 1)(r^y - 1)$ for $x, y \geq 1$.

Furthermore, $r = 1$ must be a root of odd multiplicity, since our weak-selection analysis implies a sign change in $\rho_{SH_{n,m,h}}(r) - \rho_{K_{nm+h}}(r)$ at $r = 1$. We conclude that $r = 1$ is a triple root, and that the polynomial of Eq. (65) has exactly one other positive root. This other root must be greater than 1 since we have shown $\rho_{SH_{n,m,h}}(r) < \rho_{K_{nm+h}}(r)$ for $0 < r < 1$. Therefore, $\rho_{SH_{n,m,h}}(r) < \rho_{K_{nm+h}}(r)$ only on some connected interval $(1, r^*)$.

Combining the two subcases of Case 3 shows that the Separated Hubs graph is a transient amplifier for $n \geq h + 2$. □

5.2 Fan

The Fan is the $h = 1$ case of the Separated Hubs graph; that is, $F_{n,m} = SH_{n,m,1}$. Substituting $h = 1$ into Eqs. (41) and (42) gives the effective population size for the Fan $F_{n,m}$:

$$\begin{aligned} N_{\text{eff}} &= N \\ &+ \frac{\big(m - 1 - \epsilon(nm-1)\big)\big(m(m-1)(n-2) + \epsilon(nm^2 + nm - 4m + 2) + 2\epsilon^2(nm-1)\big)}{(m - 1 + 2\epsilon)\big(m(m-1) + \epsilon(nm + 2m - 1) + \epsilon^2(nm+1)\big)}. \end{aligned} \tag{66}$$

For $\epsilon \to 0$, this becomes $N_{\text{eff}} = nm + n - 1$, which matches Eq. (43).

For arbitrary mutant fitness r, and $\epsilon \to 0$, substituting $h = 1$ into Eq. (51) gives the fixation probability

$$\rho_{F_{n,m}}(r) = \left(\frac{n(m-1)}{nm+1}\right)\left(\frac{(1 - r^{-1})(1 - r^{-(m+1)})}{(1 - r^{-(m-1)})(1 - r^{-n(m+1)})}\right). \tag{67}$$

From Theorem 3, the m-Fan is a reducer when $n = 2$, a suppressor when $n = 1$, and a transient amplifier when $n \geq 3$.

An interesting case is the 2-Fan ($m = 2$), with $r > 1$ and $n \to \infty$. In this case, we have

$$\lim_{n\to\infty} \rho_{F_{n,2}}(r) = \frac{1 - r^{-3}}{2}. \tag{68}$$

Setting this equal to the large-population limit for a complete graph, $\lim_{N\to\infty} \rho_{K_N}(r) = 1 - r^{-1}$, allows us to determine the fitness at which the graph is no longer amplifying selection. Solving

$$\frac{1 - r^{-3}}{2} = 1 - r^{-1}, \tag{69}$$

we obtain $r = 1$ or $r = (1 \pm \sqrt{5})/2$. Thus the 2-Fan, as the number of blades goes to infinity, amplifies selection on the interval $1 < r \leq \phi$, where $\phi = (1 + \sqrt{5})/2$ is the golden ratio.

5.3 Star of Islands

The Star of Islands graph $SI_{n,m,h}$ consists of $h \geq 2$ "hub" vertices and $n \geq 2$ "islands", with $m \geq 2$ vertices per island. Within the hub and each island, vertices are connected to one another with weight 1. Each hub vertex is also connected to each island vertex with weight ϵ.

5.3.1 Weak Selection

Let H represent a hub vertex and B represent any island vertex. The weighted degree and relative weighted degrees of these vertices are

$$\begin{aligned}
w_H &= h - 1 + nm\epsilon \\
w_B &= m - 1 + h\epsilon \\
\pi_H &= \frac{w_H}{hw_H + nmw_B} = \frac{h - 1 + nm\epsilon}{h(h - 1 + nm\epsilon) + nm(m - 1 + h\epsilon)} \\
\pi_B &= \frac{w_B}{hw_H + nmw_B} = \frac{m - 1 + h\epsilon}{h(h - 1 + nm\epsilon) + nm(m - 1 + h\epsilon)}.
\end{aligned}$$

Furthermore, let B and B' represent any two vertices on the same island and H and H' represent any two hub vertices. The step probabilities are:

$$\begin{aligned}
p_{BB'} &= \frac{1}{w_B} = \frac{1}{m - 1 + h\epsilon} \\
p_{BH} &= \frac{\epsilon}{w_B} = \frac{\epsilon}{m - 1 + h\epsilon} \\
p_{HB} &= \frac{\epsilon}{w_H} = \frac{\epsilon}{h - 1 + nm\epsilon} \\
p_{HH'} &= \frac{1}{w_H} = \frac{1}{h - 1 + nm\epsilon}.
\end{aligned}$$

All other step probabilities are zero.

When solving Eq. (11), there are four coalescence times to consider: $\tau_{HH'}$ for two different hub vertices, τ_{HB} for a hub and an island vertex, $\tau_{BB'}$ for two different vertices on a common island, and $\tau_{BB''}$ for two vertices on different islands.

For $\tau_{HH'}$, a walker from a hub vertex can step to any island vertex, with probability p_{HB} each and resulting coalescence time τ_{HB}. Alternatively, the walker can step to another of the remaining $h - 2$ hub vertices, with probability $p_{HH'}$ each and resulting coalescence time $\tau_{HH'}$. Together this gives

$$\tau_{HH'} = 1 + nmp_{HB}\tau_{HB} + (h - 2)p_{HH'}\tau_{HH'}. \tag{70}$$

For τ_{HB}, we have the following possibilities:

- Hub walker steps to one of $m - 1$ vertices on the same island as the other walker, with probability p_{HB} each and resulting coalescence time $\tau_{BB'}$.

- Hub walker steps to one of $(n-1)m$ vertices on a different island from the other walker, with probability p_{HB} each and resulting coalescence time $\tau_{BB''}$.
- Hub walker steps to one of $h-1$ other hub vertices, with probability $p_{HH'}$ each and resulting coalescence time τ_{HB}.
- Island walker steps to one of $m-1$ vertices on its island, with probability $p_{BB'}$ each and resulting coalescence time τ_{HB}.
- Island walker steps to one of $h-1$ other hub vertices, with probability p_{BH} each and resulting coalescence time $\tau_{HH'}$.

This gives

$$\tau_{HB} = 1 + \frac{1}{2}\Big((m-1)p_{HB}\tau_{BB'} + (n-1)mp_{HB}\tau_{BB''} + (h-1)p_{HH'}\tau_{HB} + (m-1)p_{BB'}\tau_{HB} + (h-1)p_{BH}\tau_{HH'}\Big). \quad (71)$$

For $\tau_{BB'}$, a walker can step to one of the remaining $m-2$ vertices on the same island (probability $p_{BB'}$ each, resulting coalescence time $\tau_{BB'}$) or to one of the h hub vertices (probability p_{BH} each, resulting coalescence time τ_{HB}).

$$\tau_{BB'} = 1 + (m-2)p_{BB'}\tau_{BB'} + hp_{BH}\tau_{HB}. \quad (72)$$

For $\tau_{BB''}$ a walker can then step to one of the $m-1$ vertices on its own island (probability $p_{BB'}$ each, resulting coalescence time $\tau_{BB''}$) or to one of the h hub vertices (probability p_{BH} each, resulting coalescence time τ_{BH}).

$$\tau_{BB''} = 1 + (m-1)p_{BB'}\tau_{BB''} + hp_{BH}\tau_{HB}. \quad (73)$$

Solving system Eqs. (70)–(73) and substituting into Eqs. (17) and (21) yields the effective population size for this graph. The result for arbitrary ϵ is rather cumbersome, but in the $\epsilon \to 0$ limit it simplifies to

$$\lim_{\epsilon\to 0} N_{\text{eff}} = N + \frac{(m-h)nmh\big(h(h-1) + m(m-1)(n-2)\big)}{\big(h(h-1)+m(m-1)\big)\big(h(h-1)+mn(m-1)\big)}. \quad (74)$$

Fixation probabilities for weak selection can be computed by substituting in Eq. (22). In the limit of many islands we have

$$\lim_{n\to\infty}\lim_{\epsilon\to 0} \rho' = \frac{(m-1)(h+m)}{2\big(h(h-1)+m(m-1)\big)}. \quad (75)$$

To determine the strongest amplifier of weak selection, we observe that Eq. (75) is maximized for $m = h + \sqrt{2h(h-1)}$. Substituting this maximizing value into Eq. (75), we obtain

$$\max_m \lim_{n\to\infty}\lim_{\epsilon\to 0} \rho' = \frac{-1 + h\big(2h - 3 + 2\sqrt{2h(h-1)}\big)}{-2 + 8h(h-1)}.$$

We call the right-hand side $g(h)$. The derivative of g is

$$\frac{dg}{dh} = \frac{1 + 4h^3 - 5h + (3 - 4h^2)\sqrt{2h(h-1)}}{2(h-1)(1 + 4h - 4h^2)^2}.$$

Note that since $h \geq 2$, the denominator is positive. Moreover, we have $\sqrt{2h(h-1)} \geq h$, and it follows that the numerator of $g'(h)$ is negative:

$$1 + 4h^3 - 5h + (3 - 4h^2)\sqrt{2h(h-1)} \leq 1 + 4h^3 - 5h + (3 - 4h^2)h = 1 - 2h < 0.$$

Thus $g(h)$ is decreasing in h and is maximized when $h = 2$. The largest value of ρ' therefore occurs when $h = 2$, $m = 4$ and $\rho' = \frac{9}{14}$. For comparison, the complete graph K_N has $\rho' = 1/2$ in the $N \to \infty$ limit.

5.3.2 Nonweak Selection

Here we compute the fixation probability for the Star of Islands graph in the $\epsilon \to 0$ limit. As in Section 5.1.2, we use a separation of timescales argument. Here, fixation on the hub or on the islands occurs on a fast timescale, while the spread of types between the hub and the island occurs on a slow timescale. On the slow timescale, the only relevant states are those for which the hub and islands each contain all mutants or all residents. We label such states as (M, k) or (R, k), where the first coordinate indicates the type of the hub (M or R), and the second indicate the number k of islands that have been fixed for mutants, $0 \leq k \leq n$. Dynamics on the slow timescale can be represented as a continuous-time Markov chain. The transition rate from state (M, k) to $(M, k+1)$ is given by

$$Q_{(M,k)\to(M,k+1)} = \left(\frac{(n-k)m}{nm+h}\right)\left(\frac{hr\epsilon}{m-1+hr\epsilon}\right)\left(\frac{m-1}{m}\frac{1-r^{-1}}{1-r^{-(m-1)}}\right). \tag{76}$$

The first factor on the right-hand side is the probability that, in a given time-step, a resident on an island vertex dies. The second factor is the probability that a mutant is chosen to reproduce into the vacant island vertex. The third factor is the probability that, from this initial mutant invader, the mutant type ultimately achieves fixation on this island.

Similarly, we define the three other transition rates:

$$Q_{(M,k)\to(R,k)} = \left(\frac{h}{nm+h}\right)\left(\frac{(n-k)m\epsilon}{(h-1)r + (n-k+kr)m\epsilon}\right)\left(\frac{h-1}{h}\frac{1-r}{1-r^{h-1}}\right) \tag{77}$$

$$Q_{(R,k)\to(R,k-1)} = \left(\frac{km}{nm+h}\right)\left(\frac{h\epsilon}{(m-1)r + h\epsilon}\right)\left(\frac{m-1}{m}\frac{1-r}{1-r^{m-1}}\right) \tag{78}$$

$$Q_{(R,k)\to(M,k)} = \left(\frac{h}{nm+h}\right)\left(\frac{mkr\epsilon}{h-1+(n-k+kr)m\epsilon}\right)\left(\frac{h-1}{h}\frac{1-r^{-1}}{1-r^{-(h-1)}}\right). \tag{79}$$

All other transitions are impossible, and are therefore assigned rate zero.

We discretize the above Markov chain by defining following transition probabilities, conditioned on leaving the current state, in the $\epsilon \to 0$ limit:

$$
\begin{aligned}
P_{(M,k)\to(M,k+1)} &= \lim_{\epsilon\to 0} \frac{Q_{(M,k)\to(M,k+1)}}{Q_{(M,k)\to(M,k+1)} + Q_{(M,k)\to(R,k)}} \\
&= \frac{hr^m(r^{h-1}-1)}{m(r^{m-1}-1) + hr^m(r^{h-1}-1)} \qquad (80) \\
P_{(M,k)\to(R,k)} &= \lim_{\epsilon\to 0} \frac{Q_{(M,k)\to(R,k)}}{Q_{(M,k)\to(M,k+1)} + Q_{(M,k)\to(R,k)}} \\
&= \frac{m(r^{m-1}-1)}{hr^m(r^{h-1}-1) + m(r^{m-1}-1)} \qquad (81) \\
P_{(R,k)\to(R,k-1)} &= \lim_{\epsilon\to 0} \frac{Q_{(R,k)\to(R,k-1)}}{Q_{(R,k)\to(R,k-1)} + Q_{(R,k)\to(M,k)}} \\
&= \frac{h(r^{h-1}-1)}{h(r^{h-1}-1) + mr^h(r^{m-1}-1)} \qquad (82) \\
P_{(R,k)\to(M,k)} &= \lim_{\epsilon\to 0} \frac{Q_{(R,k)\to(M,k)}}{Q_{(R,k)\to(M,k)} + Q_{(R,k)\to(M,k)}} \\
&= \frac{mr^h(r^{m-1}-1)}{h(r^{h-1}-1) + mr^h(r^{m-1}-1)}. \qquad (83)
\end{aligned}
$$

We observe that these conditional transition probabilities are independent of k.

We now follow the method of analysis that Hadjichrysanthou et al. [7] developed for the Star graph. Let $\rho_{(M,k)}$ represent the fixation probability from state (M,k), and $\rho_{(R,k)}$ the fixation probability from state (R,k). These fixation probabilities obey the recurrence relations

$$
\begin{aligned}
\rho_{(M,k)} &= P_{(M,k)\to(M,k+1)}\,\rho_{(M,k+1)} + P_{(M,k)\to(R,k)}\,\rho_{(R,k)}, \;\; 0 \le k \le n-1, \\
\rho_{(R,k)} &= P_{(R,k)\to(M,k)}\,\rho_{(M,k)} + P_{(R,k)\to(R,k-1)}\,\rho_{(R,k-1)}, \;\; 1 \le k \le n,
\end{aligned} \qquad (84)
$$

with boundary conditions $\rho_{(R,0)} = 0$ and $\rho_{(M,n)} = 1$.

The solution to recurrence relations of the form (84) was given in Ref. [7]:

$$
\begin{aligned}
\rho_{(M,i)} &= \frac{1 + \sum_{j=1}^{i-1} P_{(M,j)\to(R,j)} \prod_{k=1}^{j} \frac{P_{(R,k)\to(R,k-1)}}{P_{(M,k)\to(M,k+1)}}}{1 + \sum_{j=1}^{n-1} P_{(M,j)\to(R,j)} \prod_{k=1}^{j} \frac{P_{(R,k)\to(R,k-1)}}{P_{(M,k)\to(M,k+1)}}} \\
\rho_{(R,i)} &= \sum_{j=1}^{i} P_{(R,j)\to(M,j)}\,\rho_{(M,i)} \prod_{k=j+1}^{i} P_{(R,k)\to(R,k-1)}.
\end{aligned}
$$

In particular, the fixation probabilities starting from a single mutant are:

$$\rho_{(R,1)} = \frac{P_{(R,1)\to(M,1)}}{1 + \sum_{j=1}^{n-1} P_{(M,j)\to(R,j)} \prod_{k=1}^{j} \frac{P_{(R,k)\to(R,k-1)}}{P_{(M,k)\to(M,k+1)}}} \tag{85}$$

$$\rho_{(M,0)} = \frac{P_{(M,0)\to(M,1)}}{1 + \sum_{j=1}^{n-1} P_{(M,j)\to(R,j)} \prod_{k=1}^{j} \frac{P_{(R,k)\to(R,k-1)}}{P_{(M,k)\to(M,k+1)}}}. \tag{86}$$

Let us define

$$x = \frac{P_{(R,k)\to(R,k-1)}}{P_{(M,k)\to(M,k+1)}} = \frac{mr^{-m}(r^{m-1}-1) + h(r^{h-1}-1)}{mr^{h}(r^{m-1}-1) + h(r^{h-1}-1)}. \tag{87}$$

Substituting in Eqs. (85) and (86), we obtain

$$\rho_{(R,1)} = \frac{P_{(R,1)\to(M,1)}}{1 + \frac{x^n - x}{x-1} P_{(M,k)\to(R,k)}} \tag{88}$$

$$\rho_{(M,0)} = \frac{P_{(M,0)\to(M,1)}}{1 + \frac{x^n - x}{x-1} P_{(M,k)\to(R,k)}}. \tag{89}$$

To obtain the overall fixation probability from a single mutant (in the $\epsilon \to 0$ limit), we must consider two possibilities: (i) the mutation first arises on an island, becomes fixed on that island, and sweeps to fixation from there, or (ii) the mutant first arises on the hub, becomes fixed on the hub, and then sweeps to fixation. Putting these cases together yields:

$$\rho_{SI_{n,m,h}}(r) = \left(\frac{nm}{nm+h}\right)\left(\frac{m-1}{m}\frac{1-r^{-1}}{1-r^{-(m-1)}}\right)\rho_{(R,1)} + \left(\frac{h}{nm+h}\right)\left(\frac{h-1}{h}\frac{1-r^{-1}}{1-r^{-(h-1)}}\right)\rho_{(M,0)}.$$

Substituting from Eqs. (88) and (89), we obtain

$$\rho_{SI_{n,m,h}}(r) = \text{num}/\text{denom}, \tag{90}$$

where

$$\begin{aligned}\text{num} = {} & r^m(1-r^{-1})\left(1-r^{-(h+m)}\right) \\ & \times \Big(hr^h\left(1-r^{-(h-1)}\right)\left(nm(m-1)r^m + h(h-1)\right) \\ & \quad + mr^m\left(1-r^{-(m-1)}\right)\left(nm(m-1)+h(h-1)r^h\right)\Big),\end{aligned} \tag{91}$$

and

$$\begin{aligned}\text{denom} = {} & (nm+h)\left(h\left(1-r^{-(h-1)}\right) + mr^m\left(1-r^{-(m-1)}\right)\right) \\ & \times \Big(mr^m\left(1-r^{-(m-1)}\right)(1-x^n) + h\left(1-r^{-(h-1)}\right)\left(r^{h+m}-x^n\right)\Big).\end{aligned} \tag{92}$$

In the limit of many islands, we have

$$\lim_{n\to\infty} \rho_{SI_{n,m,h}}(r) = \begin{cases} 0 & 0 \le r \le 1 \\ \frac{(m-1)(1-r^{-1})\left(1-r^{-(m+h)}\right)}{hr^{-m}\left(1-r^{-(h-1)}\right)+m\left(1-r^{-(m-1)}\right)} & r > 1. \end{cases} \tag{93}$$

Theorem 4. *Given $m, n > 1$ and $\epsilon \to 0$, the Star of Islands is a reducer for $m = h$.*

Proof. We wish to compare fixation probabilities on the Star of Islands graph, $SI_{n,m,h}$, to those on a complete graph of equal size, K_{nm+h}, in the case $m = h$. Substituting $h = m$ into (91) and (92), and comparing to the fixation probability for K_{nm+m} from the main text, we obtain

$$\frac{\rho_{SI_{n,m,m}}(r)}{\rho_{K_{nm+m}}(r)} = \frac{\left(\dfrac{1 - r^{-(nm+m-1)}}{nm + m - 1}\right)\left(\dfrac{1 - r^{-m}}{m}\right)}{\left(\dfrac{1 - r^{-(m-1)}}{m - 1}\right)\left(\dfrac{1 - r^{-m(n+1)}}{m(n + 1)}\right)}.$$

The logarithm of this ratio can be written in terms of the function $F_r(x) = \ln\left|\frac{1-r^{-x}}{x}\right|$:

$$\ln\left(\frac{\rho_{SI_{n,m,m}}(r)}{\rho_{K_{nm+m}}(r)}\right) = F_r(nm + m - 1) + F_r(m) - F_r(m - 1) - F_r(nm + m).$$

From Lemma 1, we know that $F_r(x)$ is strictly convex in x for all $r \neq 1$. Invoking Lemma 2 with $a = m - 1$, $b = nm + m$, and $d = 1$, we see that,

$$F_r(m) + F_r(nm + m - 1) < F_r(m - 1) + F_r(nm + m), \tag{94}$$

and therefore $\ln\left(\frac{\rho_{SI_{n,m,m}}(r)}{\rho_{K_{nm+m}}(r)}\right) < 0$, for all $r \neq 1$, which proves that Star of Islands graph is a reducer for $m = h$. □

References

[1] Allen B, Tarnita CE. Measures of success in a class of evolutionary models with fixed population size and structure. Journal of Mathematical Biology. 2014;68(1-2):109–143.

[2] Allen B, Lippner G, Chen YT, Fotouhi B, Momeni N, Yau ST, et al. Evolutionary dynamics on any population structure. Nature. 2017;544(7649):227–230.

[3] Allen B, McAvoy A. A mathematical formalism for natural selection with arbitrary spatial and genetic structure. Journal of Mathematical Biology. 2018;doi:10.1007/s00285-018-1305-z.

[4] Maciejewski W. Reproductive value in graph-structured populations. Journal of Theoretical Biology. 2014;340:285–293.

[5] Allen B, Sample C, Dementieva Y, Medeiros RC, Paoletti C, Nowak MA. The Molecular Clock of Neutral Evolution Can Be Accelerated or Slowed by Asymmetric Spatial Structure. PLOS Computational Biology. 2015;11(2):e1004108. doi:10.1371/journal.pcbi.1004108.

[6] Descartes R. La Géométrie; 1637.

[7] Hadjichrysanthou C, Broom M, Rychtář J. Evolutionary games on star graphs under various updating rules. Dynamic Games and Applications. 2011;1(3):386.
