# Supplementary material for: Transient amplifiers of selection and reducers of fixation for death-Birth updating on graphs
Source: PLoS Comput Biol. 2020 Jan 17;16(1):e1007529. doi: 10.1371/journal.pcbi.1007529 (PMC6968840; doi:10.1371/journal.pcbi.1007529)
Supplement: Attachment — Submitted filename: PCOMPBIOL-D-19-00886_reviewer.pdf [file pcbi.1007529.s004.pdf]

PLOS Computational Biology

Transient amplifiers of selection and reducers of fixation for death-Birth updating on graphs

--Manuscript Draft--

**Manuscript Number:**	PCOMPBIOL-D-19-00886
**Full Title:**	Transient amplifiers of selection and reducers of fixation for death-Birth updating on graphs
**Short Title:**	Transient amplifiers and reducers for death-Birth updating
**Article Type:**	Research Article
**Keywords:**	evolution; Fixation probability; Graph; Amplifier; Suppressor
**Abstract:**	The spatial structure of an evolving population affects which mutations become fixed. Some structures amplify selection, increasing the likelihood that beneficial mutations become fixed while deleterious mutations do not. Other structures suppress selection, reducing the effect of fitness differences and increasing the role of random chance. This phenomenon can be modeled by representing spatial structure as a graph, with individuals occupying vertices. Births and deaths occur stochastically, according to a specified update rule. We study death-Birth updating: An individual is chosen to die and then its neighbors compete to reproduce into the vacant spot. Previous numerical experiments suggested that amplifiers of selection for this process are either rare or nonexistent. We introduce a perturbative method for this problem for weak selection regime, meaning that mutations have small fitness effects. We show that fixation probability under weak selection can be calculated in terms of the coalescence times of random walks. This result leads naturally to a new definition of effective population size. Using this and other methods, we uncover the first known examples of transient amplifiers of selection (graphs that amplify selection for a particular range of fitness values) for the death-Birth process. We also exhibit new families of ``reducers of fixation", which decrease the fixation probability of all mutations, whether beneficial or deleterious.
**Additional Information:**	
**Question**	**Response**
**Financial Disclosure**<br><br>Enter a financial disclosure statement that describes the sources of funding for the work included in this submission. Review the submission guidelines for detailed requirements. View published research articles from *PLOS Computational Biology* for specific examples.<br><br>This statement is required for submission and **will appear in the published article** if the submission is accepted. Please make sure it is accurate.	BA, CS, LB, RJ, JK, and PS were supported by the National Science Foundation Award #1715315.<br><br>https://nsf.gov/awardsearch/showAward?AWD_ID=1715315&HistoricalAwards=false<br><br>The funders had no role in study design, data collection and analysis, decision to publish, or preparation of the manuscript.

Powered by Editorial Manager® and ProduXion Manager® from Aries Systems Corporation

**Unfunded studies**<br>Enter: *The author(s) received no specific funding for this work.*<br><br>**Funded studies**<br>Enter a statement with the following details:<br>• Initials of the authors who received each award<br>• Grant numbers awarded to each author<br>• The full name of each funder<br>• URL of each funder website<br>• Did the sponsors or funders play any role in the study design, data collection and analysis, decision to publish, or preparation of the manuscript?<br>• **NO** - Include this sentence at the end of your statement: *The funders had no role in study design, data collection and analysis, decision to publish, or preparation of the manuscript.*<br>• **YES** - Specify the role(s) played.<br><br>* typeset	
**Competing Interests**<br><br>Use the instructions below to enter a competing interest statement for this submission. On behalf of all authors, disclose any competing interests that could be perceived to bias this work—acknowledging all financial support and any other relevant financial or non-financial competing interests.<br><br>This statement **will appear in the published article** if the submission is accepted. Please make sure it is accurate. View published research articles from *PLOS Computational Biology* for specific examples.	The authors have declared that no competing interests exist

Powered by Editorial Manager® and ProduXion Manager® from Aries Systems Corporation

**NO authors have competing interests**<br><br>Enter: *The authors have declared that no competing interests exist.*<br><br>**Authors with competing interests**<br><br>Enter competing interest details beginning with this statement:<br><br>*I have read the journal's policy and the authors of this manuscript have the following competing interests: [insert competing interests here]*<br><br>* typeset	
**Data Availability**<br><br>Authors are required to make all data underlying the findings described fully available, without restriction, and from the time of publication. PLOS allows rare exceptions to address legal and ethical concerns. See the PLOS Data Policy and FAQ for detailed information.<br><br>A Data Availability Statement describing where the data can be found is required at submission. Your answers to this question constitute the Data Availability Statement and **will be published in the article**, if accepted.<br><br>**Important:** Stating 'data available on request from the author' is not sufficient. If your data are only available upon request, select 'No' for the first question and explain your exceptional situation in the text box.<br><br>Do the authors confirm that all data underlying the findings described in their manuscript are fully available without restriction?	Yes - all data are fully available without restriction
**Describe where the data may be found in full sentences. If you are copying our sample text, replace any instances of XXX with the appropriate details.**	All relevant data are within the manuscript and its Supporting Information files.

• If the data are **held or will be held in a public repository**, include URLs, accession numbers or DOIs. If this information will only be available after acceptance, indicate this by ticking the box below. For example: *All XXX files are available from the XXX database (accession number(s) XXX, XXX.*).<br>• If the data are all contained **within the manuscript and/or Supporting Information files**, enter the following: *All relevant data are within the manuscript and its Supporting Information files.*<br>• If neither of these applies but you are able to provide **details of access elsewhere**, with or without limitations, please do so. For example:<br><br>*Data cannot be shared publicly because of [XXX]. Data are available from the XXX Institutional Data Access / Ethics Committee (contact via XXX) for researchers who meet the criteria for access to confidential data.*<br><br>*The data underlying the results presented in the study are available from (include the name of the third party and contact information or URL).*<br>• This text is appropriate if the data are owned by a third party and authors do not have permission to share the data.<br><br>* typeset	
Additional data availability information:	

EMMANUEL COLLEGE

May 29, 2019

Ruth Nussinov, Jason Pappin, Phillip E. Bourne
Editors-in-Chief
PLOS Computational Biology

Dear Drs. Nussinov, Pappin, and Bourne,

We are pleased to submit our manuscript, entitled **"Transient amplifiers of selection and reducers of fixation for death-Birth updating on graphs"** by Benjamin Allen et al., to be considered for publication in *PLOS Computational Biology*.

The spatial structure of a population can either amplify or suppress the effects of natural selection. For over a decade, these effects have been the subject of intensive mathematical investigation, using the framework of evolutionary graph theory. Most of these works consider only the Birth-death (Bd) update rule; the death-Birth (dB) rule is a natural alternative that has received less attention.

A previous numerical investigation (Hindersin and Traulsen, *PLOS Comp Biol*, 2015) found no amplifiers of selection for dB updating among thousands of small graphs. This raised the question: Are amplifiers for dB impossible, or merely rare?

We report here that amplifiers for dB are indeed possible, as long as they are *transient* (amplifying only for a certain range of mutant fitness values). We exhibit several families of transient amplifiers. We also identify families of *reducers*, which lessen the fixation probability of both beneficial and deleterious mutations.

Our work brings new theoretical and computational tools—weak selection expansion and coalescing random walks—to bear on this question. These tools lead to new theoretical results, as well as to a new, natural definition of the effective population size of a graph.

We suggest that this work be considered as a companion to the manuscript **"Limits on amplifiers of natural selection under death-Birth updating"** by Josef Tkadlec et al., also submitted to *PLOS Computational Biology*. The two manuscripts complement each other: We show the existence of (transient) amplifiers, while Tkadlec et al. prove limits on how strong such amplifiers can be. Although we derived our results independently of each other, we have integrated references to each other's work in our manuscripts.

As a handling editor we suggest Arne Traulsen, who is a leading expert on the problem of amplifiers and suppressors. As reviewers, we suggest Mark Broom (City University of London), Laura Hindersin (Max Planck Institute for Evolutionary Biology), Fernando Cuesta (Universidade de Santiago de Compostela), Jan Rychtar (University of North Carolina-Greensboro), or Christoph Hauert (University of British Coliumbia).

We thank you for your consideration of our manuscript.

EMMANUEL COLLEGE

Peace,

Benjamin Allen
Associate Professor of Mathematics
Emmanuel College

Manuscript Click here to download Manuscript Main.pdf

Transient amplifiers of selection and reducers of fixation for death-Birth updating on graphs

Benjamin Allen[1,2,*], Christine Sample[1], Robert Jencks[1], James Withers[1], Patricia Steinhagen[1], Lori Brizuela[1], Joshua Kolodny[1], Darren Parke[1], Gabor Lippner[3], Yulia A. Dementieva[1]

1 Emmanuel College, Boston, MA, USA
2 Program for Evolutionary Dynamics, Harvard University, Cambridge, MA, USA
3 Department of Mathematics, Northeastern University, Boston, MA, USA

*allenb@emmanuel.edu

Abstract

The spatial structure of an evolving population affects which mutations become fixed. Some structures amplify selection, increasing the likelihood that beneficial mutations become fixed while deleterious mutations do not. Other structures suppress selection, reducing the effect of fitness differences and increasing the role of random chance. This phenomenon can be modeled by representing spatial structure as a graph, with individuals occupying vertices. Births and deaths occur stochastically, according to a specified update rule. We study *death-Birth* updating: An individual is chosen to die and then its neighbors compete to reproduce into the vacant spot. Previous numerical experiments suggested that amplifiers of selection for this process are either rare or nonexistent. We introduce a perturbative method for this problem for weak selection regime, meaning that mutations have small fitness effects. We show that fixation probability under weak selection can be calculated in terms of the coalescence times of random walks. This result leads naturally to a new definition of effective population size. Using this and other methods, we uncover the first known examples of transient amplifiers of selection (graphs that amplify selection for a particular range of fitness values) for the death-Birth process. We also exhibit new families of "reducers of fixation", which decrease the fixation probability of all mutations, whether beneficial or deleterious.

Author summary

Natural selection is often thought of as "survival of the fittest", but random chance plays a significant role in which mutations persist and which are eliminated. The balance of selection versus randomness is affected by spatial structure—how individuals are arranged within their habitat. Some structures amplify the effects of selection, so that only the fittest mutations are likely to persist. Others suppress the effects of selection, making the survival of genes primarily a matter of random chance. We study this question using a mathematical model called the "death-Birth process". Previous studies have found that spatial structure rarely, if ever, amplifies selection for this process. Here we report that spatial structure can indeed amplify selection, at least for mutations with small fitness effects. We also identify structures that reduce the spread of any new mutation, whether beneficial or deleterious. Our work introduces new mathematical techniques for assessing how population structure affects natural selection.

Introduction

Spatial population structure has a variety of effects on natural selection [1–5]. These effects can be studied mathematically by representing spatial structure as a graph [3]. The vertices represent individuals, and the edges indicate spatial relationships between them. This modeling approach, known as evolutionary graph theory, has illuminated the effects of spatial structure on the rate of genetic change [6], the balance of selection versus neutral drift [3, 7, 8], and the evolution of cooperation and other social behaviors [4, 5, 9–14].

Here we focus on how spatial structure affects fixation probability—the p[illegible]ility that a new mutation will spread throughout the population, depending on it[illegible]ct on fitness. Previous work [3, 7, 8, 15–26] has shown that some graphs act as *amplifiers* of selection, increasing the fixation probability of beneficial mutations, while reducing that of deleterious mutations. Other graphs act as *suppressors* of selection, increasing the fixation probability of deleterious mutations and reducing that of beneficial mutations. Over time, a population that is structured as an amplifier will more rapidly accrue beneficial mutations, whereas one structured as a suppressor will experience greater effects of random drift.

To be precise, the terms amplifier and suppressor cannot be ascribed solely to a graph itself. Fixation probabilities also depend on the *update rule:* the scheme by which births and deaths are determined. The majority of works on amplifiers and suppressors use *Birth-death (Bd)* updating: An individual is selected to reproduce proportionally to fitness, and its offspring replaces a uniformly-chosen neighbor. A minority of works [17, 22, 27, 28] have considered *death-Birth (dB)* updating: A uniformly-chosen individual dies, and a neighbor is chosen proportionally to fitness to reproduce into the vacancy. (Following Hindersin and Traulsen [22], we use uppercase letters for a demographic step that is affected by fitness, and lowercase letter[illegible] a step that is fitness-independent.) Interestingly, the choice of update rule has[illegible]rked effect on fixation probabilities. For example, the Star graph (Fig. 1B) is an amplifier of selection for Bd updating [3, 16], but a suppressor for dB updating [17].

A recent numerical investigation [22] of thousands of random graphs up to size 14 found no amplifiers of selection for death-Birth updating. This suggests that amplifiers for dB are either nonexistent or rare, at least among small graphs. This work also identified a graph (the cycle; Fig. 1C) that, for dB updating, reduces fixation probabilities for *all* mutations that affect fitness, whether beneficial or deleterious. The cycle is therefore neither an amplifier nor a suppressor; it might instead be called a "reducer of fixation", in that it preserves the resident wild-type regardless of fitness effects. A follow-up work [29] identified other reducers of fixation.

Here we investigate fixation probabilities for death-Birth updating on graphs, using a variety of analytical and numerical methods. We develop a weak-selection approach to this question, based on coalescing random walk methods [30, 31] that were previously used to study evolutionary games on graphs [5, 10, 14]. Weak selection means that the fitness of the mutant is close to that of the resident; i.e., the mutation is either slightly beneficial or slightly deleterious. Unlike earlier numerical methods [22, 25, 32], the weak-selection method can be performed in polynomial time, allowing for efficient identification of amplifiers and suppressors of weak selection. We apply this method to several graph families and random graph models. We also compute fixation probabilities for arbitrary mutant fitness (beyond weak selection) for these graph families.

We find, contrary to the expectation set by previous numerical experiments [22], that amplifiers, of a sort, do exist for death-Birth updating. Specifically, we exhibit several families of *transient amplifiers*, which amplify selection only for a certain range of mutant fitness values. We also uncover new examples of reducers of fixation.

Our weak-selection method also leads to new theoretical results. First, the form of

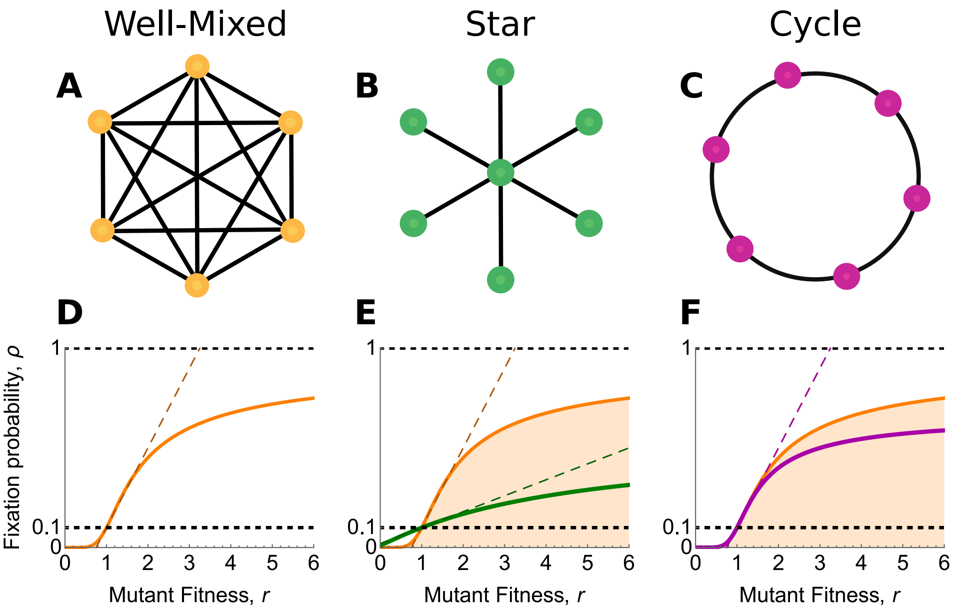

Fig 1. Fixation probabilities for constant selection on graphs. (A) The complete graph represents a well-mixed population. **(B)** The star graph consists of one hub vertex connected to n leaf vertices. This star is a suppressor of selection for death-Birth updating [17]. **(C)** The cycle, a regular graph of degree 2, is a reducer of fixation: the fixation probability of any mutant type of fitness $r \neq 1$ is smaller than it would be in the well-mixed case [22]. Panels **(D)**–**(F)** plot fixation prob[illegible] versus mutant fitness for the respective graphs, with the well-mixed case (orange [illegible]) shown for comparison. Dashed lines show the linear approximation to fixation probability at $r = 1$. These approximations are accurate for weak selection ($r \approx 1$) and can be computed from coalescence times using Eqs. (5)–(9).

our expression for fixation probability suggests a new definition of effective population size, with intriguing connections to previous definitions [15, 33–39]. Second, we show that for *isothermal* graphs—which have the same edge weight sum at each vertex—the fixation probability coincides, under weak selection, with that of a well-mixed population. This result is reminiscent of the Isothermal Theorem of Lieberman et al. [3], which applies to Bd updating (see also Refs. [27, 28]). However, whereas the original Isothermal Theorem is valid for any strength of selection, our new result applies only to weak selection. Third, we show that fixation probabilities under weak selection can be well-approximated using only the first two moments of the degree distribution. This approximation helps explain why amplifiers of selection (even transient ones) are rare for dB updating.

Model

We study an established model of natural selection on graphs [3, 7, 8, 15–28, 40–42]. Spatial structure is represented as a connected, weighted, undirected graph G. Joining each pair of vertices i and j is an edge of weight $w_{ij} \geq 0$, with $w_{ij} = w_{ji}$ since G is undirected. We exclude the possibility of self-loops by setting $w_{ii} = 0$ for each vertex i. The size of the graph, which is also the population size, is denoted N.

Each vertex houses a single haploid individual. Individuals can be of mutant or resident (wild-) type. Mutants have fitness $r > 0$, while the fitness of the resident type is set to 1. Advantageous mutants have $r > 1$, while deleterious mutants have $r < 1$. The case $r = 1$ describes neutral drift, for which the mutation has no fitness effect. This model describes *constant selection*, in that the fitnesses of the competing types do not vary with the current population state.

Selection proceeds according to the death-Birth (dB) update rule [4, 17, 43]. First, an individual is selected uniformly at random for death, creating a vacant vertex. Then, a neighbor of the vacant vertex is chosen to reproduce, with probability proportional to (fitness) × (edge weight to the vacant vertex). The new offspring fills the vacancy, inheriting the type of the parent.

As an initial state, we suppose that a single mutant is introduced, at a vertex chosen uniformly at random, in a population otherwise composed of residents. We define the mutation's *fixation probability* as the expected probability that a state of all mutants is reached from this initial condition. The fixation probability of a mutation of fitness r on a graph G is denoted $\rho_G(r)$.

The baseline case of a well-mixed population is represented by the complete graph K_N of size N (Fig 1A). For dB updating on K_N, a mutant of fitness r has fixation probability [22, 27]

$$\rho_{K_N}(r) = \frac{N-1}{N} \frac{1-r^{-1}}{1-r^{-(N-1)}}. \tag{1}$$

We characterize the effects of graph structure on fixation probabilities using the following definitions:

Definition. Let G be a graph of size N. Then G is

- An *amplifier of selection* if $\rho_G(r) < \rho_{K_N}(r)$ for $0 < r < 1$ and $\rho_G(r) > \rho_{K_N}(r)$ for $r > 1$.
- A *suppressor of selection* if $\rho_G(r) > \rho_{K_N}(r)$ for $0 < r < 1$ and $\rho_G(r) < \rho_{K_N}(r)$ for $r > 1$.
- A *transient amplifier of selection* if there is some $r^* > 1$ such that $\rho_G(r) < \rho_{K_N}(r)$ for $0 < r < 1$ and for $r > r^*$, and $\rho_G(r) > \rho_{K_N}(r)$ for $1 < r < r^*$.

- A *reducer of fixation* if $\rho_G(r) < \rho_{K_N}(r)$ for all $r \neq 1$.

For example, the star graph S_n with n leaves (population size $N = n + 1$; Fig. 1B) is a suppressor of selection for dB updating [17], with fixation probability [44]

$$\rho_{S_n}(r) = \frac{(N-1)r + 1}{N(r+1)} \left(\frac{1}{N} + \frac{r}{N + 2r - 2} \right). \tag{2}$$

The cycle C_N is a reducer of fixation for dB updating [22], with fixation probability [27]

$$\rho_{C_N}(r) = \frac{2(r-1)}{3r - 1 + r^{-(N-1)} - 3r^{-(N-2)}}. \tag{3}$$

Other examples of reducers were identified by Hindersin et al. [29], who called them "suppressors of evolution"; we prefer "reducers of fixation" to avoid confusion with suppressors of selection.

A companion work [45] proves that there are no (non-transient) amplifiers of selection for dB updating. Transient amplifiers of selection were previously known for Bd updating [18] but not for dB updating. For Bd updating, there are some graphs that do not fit any of the above definitions, but alternate between amplification and suppression (i.e, $\rho_G(r) > \rho_{K_N}(r)$ on a disconnected set of r-values) [26]; such examples have not been discovered for dB updating.

Results

Fixation probability under weak selection

Fixation probabilities on graphs can be difficult to compute. Current numerical methods [21,22,25,32] involve solving a system of $\mathcal{O}(2^N)$ equations to compute fixation probabilities on a given graph of size N. For this reason, previous analyses have focused on small graphs [22,25,26,32,41,42] and/or graphs with a high degree of symmetry [3,7,16,18–20,23,24].

One way to mitigate these difficulties is to focus on *weak selection*, which is the regime $r \approx 1$. Weak selection can be studied as a perturbation of neutral drift ($r = 1$). This approach has been fruitfully applied to population genetics [46–48] and evolutionary game theory [4,5,10,11,14,43,49], but so far has not been applied to models of constant selection on graphs.

To implement weak selection for our model, we write the fitness of the mutant as $r = 1 + \delta$, with δ representing the mutation's selection coefficient. We consider the first-order Taylor expansion of the fixation probability, $\rho_G(1+\delta)$, at $\delta = 0$. For the complete graph, Taylor expansion of Eq. (1) yields

$$\rho_{K_N}(1+\delta) = \frac{1}{N} + \delta \frac{N-2}{2N} + \mathcal{O}(\delta^2). \tag{4}$$

For an arbitrary weighted, connected graph, we apply a method developed by Allen et al. [5] to calculate fixation probabilities under weak selection. This method uses *coalescing random walks*, which trace the co-ancestry of given individuals backwards in time to their most recent common ancestor.

Each individual's ancestry is represented as a random walk on G. These random walks are defined by the step probabilities $p_{ij} = w_{ij}/w_i$, where $w_i = \sum_{j \in G} w_{ij}$ is the *weighted degree* of vertex i. Importantly, p_{ij} is also equal to the conditional probability, under neutral drift ($r = 1$), that j reproduces, given that i is replaced. Random walks on G have a stationary distribution, in which the probability of being at vertex i is equal to its relative weighted degree, $\pi_i = w_i / \left(\sum_{j \in G} w_j \right)$.

To represent the co-ancestry of two individuals, we consider a pair of random walkers. At each time-step, one of the two walkers is chosen (with equal probability) to take a step. The point at which the two walkers meet (coalesce) represents the most recent common ancestor. We let τ_{ij} denote the expected time to coalescence from initial vertices i and j. These coalescence times can be determined from the following system of equations [5, 50]:

$$\tau_{ij} = \begin{cases} 0 & i = j \\ 1 + \frac{1}{2}\sum_{k \in G} \left(p_{ik}\tau_{jk} + p_{jk}\tau_{ik}\right) & i \neq j. \end{cases} \tag{5}$$

We also define the *remeeting time* τ_i from vertex i as the expected time for two random walkers from vertex i to rejoin each other. Remeeting times are related to coalescence times by

$$\tau_i = 1 + \sum_{j \in G} p_{ij}\tau_{ij}, \tag{6}$$

and obey the identity [5]

$$\sum_{i \in G} \pi_i^2 \tau_i = 1. \tag{7}$$

Applying the properties of coalescence times, we prove in S1 Appendix that fixation probability on an arbitrary (weighted, undirected, connected) graph G can be expanded under weak selection as

$$\rho_G(1+\delta) = \frac{1}{N} + \delta\frac{N_{\text{eff}} - 2}{2N} + \mathcal{O}(\delta^2), \tag{8}$$

where N_{eff} is the *effective population size* of G, which we define as

$$N_{\text{eff}} = \sum_{i \in G} \pi_i \tau_i. \tag{9}$$

This definition of effective population size is distinct from, but closely related to, previous definitions [15, 33–39], as we review in the Discussion.

Comparing the first-order terms in Eqs. (8) and (4) provides a criterion for the effects of graph structure on fixation probabilities under weak selection:

Definition. Let G be a graph of size N. We say G is

- An *amplifier of weak selection* if $N_{\text{eff}} > N$,
- A *suppressor of weak selection* if $N_{\text{eff}} < N$.

An amplifier (respectively, suppressor) of weak selection is guaranteed to amplify (respectively, suppress) selection for all r sufficiently close to 1. Form[illegible]f G is an amplifier of weak selection, there exist a, b with $0 \leq a < 1 < b \leq \infty$ such that $\rho_G(r) < \rho_{K_N}(r)$ for $a < r < 1$ and $\rho_G(r) > \rho_{K_N}(r)$ for $1 < r < b$. Likewise, if G is a suppressor of weak selection, there exist a, b with $0 \leq a < 1 < b \leq \infty$ such that $\rho_G(r) > \rho_{K_N}(r)$ for $a < r < 1$ and $\rho_G(r) < \rho_{K_N}(r)$ for $1 < r < b$.

As an example, solving Eq. (5) for the star graph S_n, and applying Eqs. (6) and (9), we obtain $\tau_H = \tau_L = N_{\text{eff}} = 4n/(n+1)$. Since the star graph has size $N = n+1$, we find that the star is a suppressor of weak selection for all $n \geq 2$. Substituting in Eq. (8), we obtain

$$\rho_G(1+\delta) = \frac{1}{N} + \delta\frac{N-2}{N^2} + \mathcal{O}(\delta^2), \tag{10}$$

which agrees with the Taylor expansion of Eq. (2).

Weak-selection Isothermal Theorem

A particularly interesting result arises in the special case of *isothermal* graphs. An unweighted graph G is isothermal if each vertex has the same weighted degree w_i, or equivalently, if $\pi_i = 1/N$ for each $i \in G$. The *Isothermal Theorem* [3] states that, for Bd updating, an isothermal graph has the same fixation probabilities as a well-mixed population of the same size, for all values of r and all starting configurations of mutants. However, the corresponding statement for dB updating is false [27, 28]. For example, the cycle (Fig. 1C) is isothermal, but its fixation probabilities, as given by Eq. (3), differ from those of a well-mixed population, given by Eq. (1).

Here we show that a weak-selection version of the isothermal theorem holds for death-Birth updating. For an isothermal graph G, Eqs. (7) and (9) give

$$N_{\text{eff}} = \sum_{i \in G} \left(\frac{1}{N}\right) \tau_i = N \sum_{i \in G} \left(\frac{1}{N^2}\right) \tau_i = N \sum_{i \in G} \pi_i^2 \tau_i = N. \tag{11}$$

Combining with Eq. (8), we arrive at the following result:

Theorem (Weak-selection Isothermal Theorem for dB Updating)**.** *Let G be a weighted, undirected, connected isothermal graph of size $N \geq 2$ with no self-loops. Then for dB updating, fixation probabilities on G coincide with those on the complete graph K_N to first order in the selection coefficient δ:*

$$\rho_G(1+\delta) = \rho_{K_N}(1+\delta) + \mathcal{O}(\delta^2). \tag{12}$$

In other words, if G is isothermal, then the plot of $\rho_G(r)$ is tangent to that of $\rho_{K_N}(r)$ at $r = 1$. This implies that isothermal graphs are neither amplifiers nor suppressors of weak selection. For example, the cycle C_N (Fig. 1C), is isothermal, and therefore the plots of $\rho_{C_N}(r)$ and $\rho_{K_N}(r)$ are tangent at $r = 1$ (Fig. 1F). However, these plots do not coincide beyond $r = 1$; instead, $\rho_{C_N}(r) < \rho_{K_N}(r)$ for all $r \neq 1$ [32], meaning that the cycle is a reducer of fixation.

Examples

We now introduce three example families of graphs, which can behave as transient amplifiers, suppressors, or reducers, depending on the parameter values. We analyze these graphs both for weak and nonweak selection. Our results are summarized in Table 1; detailed calculations and proofs are presented in S1 Appendix.

Table 1. Results for example graphs

Example	Case	Classification
Separated Hubs* ($\epsilon \to 0$)	$n \leq h$	Suppressor
	$n = h+1$	Reducer
	$n \geq h+2$	Transient amplifier
Star of Islands ($\epsilon \to 0$)	$m \leq h-1$	Suppressor**
	$m = h$	Reducer
	$m \geq h+1$	Transient amplifier**

* The Fan is the $h = 1$ case of separated hubs.
** Proven only for weak selection (other cases are proven for arbitrary selection strength).

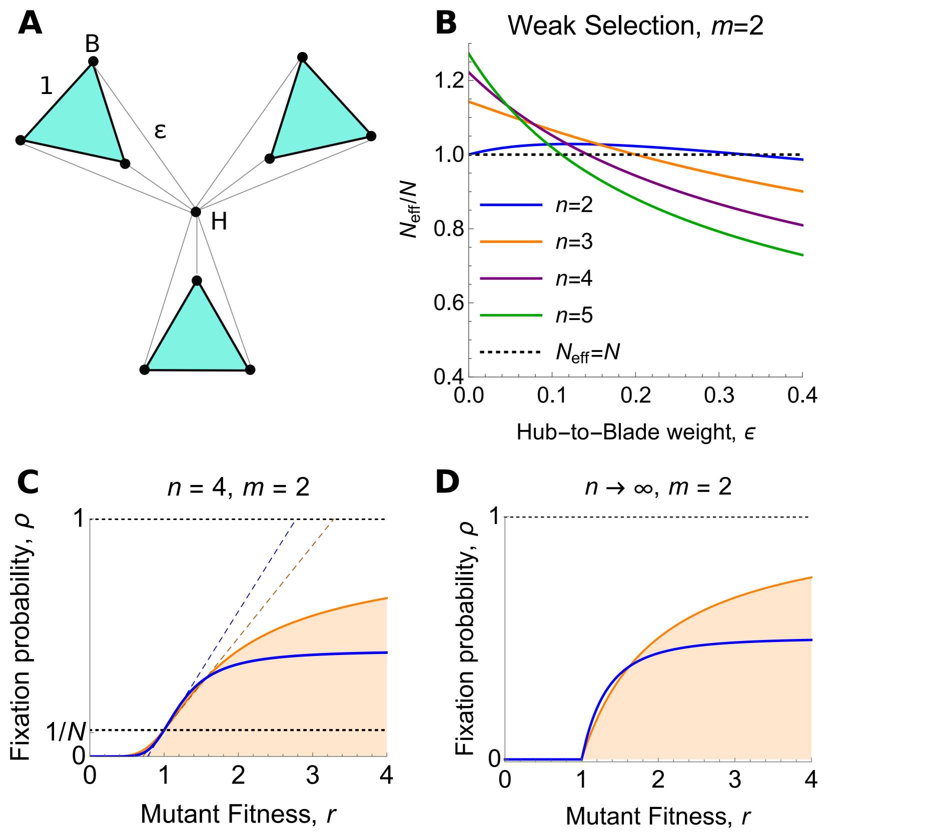

Fig 2. The Fan (A) The Fan, $F_{n,m}$, consists of one hub and $n \geq 2$ "blades", with $m \geq 2$ vertices per blade. Edge weights are as shown. The case $n = m = 3$ is pictured. **(B)** The ratio of effective versus actual population size, plotted against the hub-to-blade edge weight ϵ, for $m = 2$ vertices per blade. For $n = 2$ blades, the Fan is an amplifier of weak selection for $0 < \epsilon < 1/3$, but becomes a reducer in the $\epsilon \to 0$ limit. For $n \geq 3$, the Fan is a transient amplifier for sufficiently small ϵ, including the $\epsilon \to 0$ limit. **(C)** Fixation probability for $F_{4,2}$ (blue curve), plotted against mutant fitness r, in the $\epsilon \to 0$ limit, according to Eq. (14). The orange curve shows the corresponding well-mixed population result, Eq. (1), for comparison. Dotted lines show the corresponding weak-selection results (i.e. the linear approximation at $r = 1$), according to Eqs. (4),(8), and (13). **(D)** In the $n \to \infty$ limit, fixation probability is given by Eq. (14), and the Fan is an amplifier for $1 < r < (1 + \sqrt{5})/2$.

Fan

The Fan, $F_{n,m}$, (Fig. 2) has one hub and $n \geq 2$ blades. Each blade contains $m \geq 2$ vertices, for a total of $N = nm + 1$ vertices. Each blade vertex is joined to the hub by an edge of weight $\epsilon > 0$, and is joined to each other vertex on the same blade by an edge of weight 1. The Fan is isothermal when $\epsilon = (m-1)/(nm-1)$.

Applying our weak-selection method, we find that the Fan has effective population size

$$N_{\text{eff}} = \frac{mn(mn + 4\epsilon - 1)(m(mn + \epsilon - 1) - \epsilon)}{(m-1)\epsilon^2(mn+1) + \epsilon(m(n+2) - 1)(mn-1) + m(mn-1)^2}. \quad (13)$$

Comparing to $N = nm + 1$, we find that the Fan amplifies weak selection for all $0 < \epsilon < (m-1)/(nm-1)$ (Fig. 2B). An interesting behavior occurs in the limit $\epsilon \to 0$: For $n \geq 3$ blades the Fan amplifies weak selection ($\lim_{\epsilon \to 0} N_{\text{eff}} > N$), but for $n = 2$ blades the Fan neither amplifies nor suppresses weak selection ($\lim_{\epsilon \to 0} N_{\text{eff}} = N$). The strongest amplifier of weak selection (largest N_{eff}/N) occurs for $m = 2$ and first $\epsilon \to 0$ and then $n \to \infty$; in this case, $N_{\text{eff}}/N \to 3/2$.

Moving beyond weak selection, we calculate the fixation probability for a mutation of arbitrary fitness $r > 0$, in the $\epsilon \to 0$ limit:

$$\rho_{F_{n,m}}(r) = \frac{n(m-1)\left(1 - r^{-1}\right)\left(1 - r^{-(m+1)}\right)}{(mn+1)\left(1 - r^{-(m-1)}\right)\left(1 - r^{-n(m+1)}\right)}. \quad (14)$$

We prove in S1 Appendix that, in the $\epsilon \to 0$ limit, the Fan is a reducer of fixation for $n = 2$ and a transient amplifier of selection for all $n \geq 3$.

Separated Hubs

Our next examples generalize the Fan graph in two different ways. First, we suppose that there are multiple hub vertices, which are not connected to each other. The resulting graph, which we call the *Separated Hubs* graph, $SH_{n,m,h}$, has $h \geq 1$ hub vertices, $n \geq 2$ blades, and $m \geq 2$ vertices per blade (Fig. 3). Vertices on the same blade are connected by edges of weight 1, and each blade vertex is connected to each hub by an edge of weight ϵ. No other edges are present. The Fan is the $h = 1$ case of Separated Hubs.

The weak-selection results for arbitrary ϵ are rather cumbersome, but in the $\epsilon \to 0$ limit they simplify to

$$N_{\text{eff}} = nm + n - 1. \quad (15)$$

Interestingly, in this limit, the effective population size is independent of the number h of hubs. Comparing Eq. (15) to the population size, $N = nm + h$, we observe that the Separated Hubs graph (in the $\epsilon \to 0$ limit) is a suppressor of weak selection for $n \leq h$ and an amplifier of weak selection for $n \geq h + 2$. Again, the strongest amplifier of weak selection occurs for $m = 2$ and first $\epsilon \to 0$ and then $n \to \infty$, leading to $N_{\text{eff}}/N \to 3/2$. The strongest suppressor of weak selection (smallest N_{eff}/N) occurs for first $\epsilon \to 0$ and then $h \to \infty$, leading to $N_{\text{eff}}/N \to 0$.

Beyond weak selection, we compute the fixation probability for ar[illegible]y $r > 0$ in the limit $\epsilon \to 0$:

$$\rho_{SH_{n,m,h}}(r) = \frac{n(m-1)(1 - r^{-1})\left(1 - r^{-(m+1)}\right)}{(mn+h)\left(1 - r^{-(m-1)}\right)\left(1 - r^{-n(m+1)}\right)}. \quad (16)$$

In the limit of many blades, we obtain

$$\lim_{n \to \infty} \rho_{SH_{n,m,h}}(r) = \begin{cases} 0 & 0 \leq r \leq 1 \\ \frac{m-1}{m} \frac{(1-r^{-1})(1-r^{-(m+1)})}{1-r^{-(m-1)}} & r > 1. \end{cases} \quad (17)$$

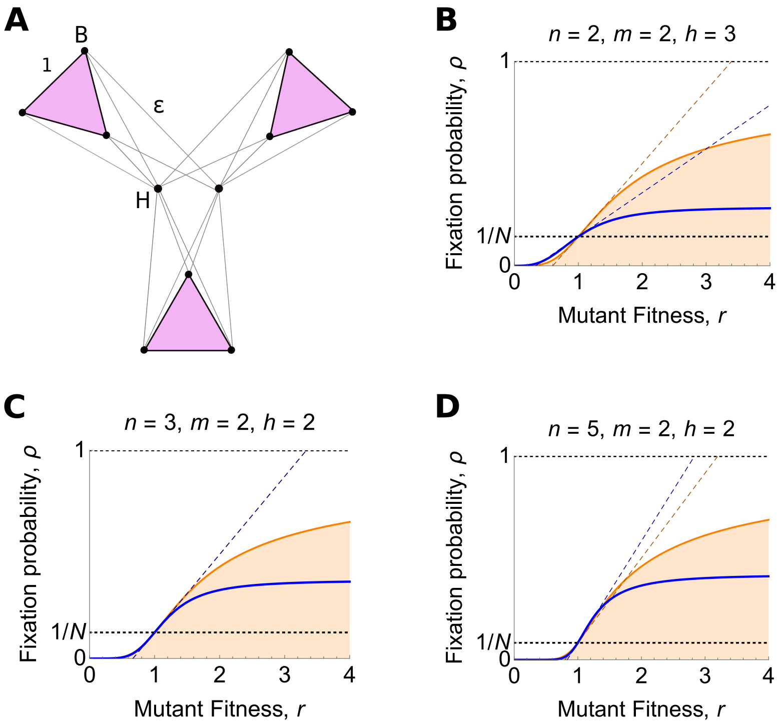

Fig 3. Separated Hubs (A) The Separated Hubs graph consists of $h \geq 1$ hubs and $n \geq 1$ blades, with $m \geq 2$ vertices per blade. Edge weights are as shown. **(B)–(D)** Blue curves show fixation probability, Eq. (16), plotted against mutant fitness r, in the $\epsilon \to 0$ limit. Blue dotted lines show the weak selection result, Eqs. (8) and (15). The orange curve and dotted line show the corresponding well-mixed population results, Eqs. (1) and (4), for comparison. The Separated Hubs graph is (B) a suppressor for $n \leq h$, (C) a reducer for $n = h + 1$, and (D) a transient amplifier for $n \geq h + 2$.

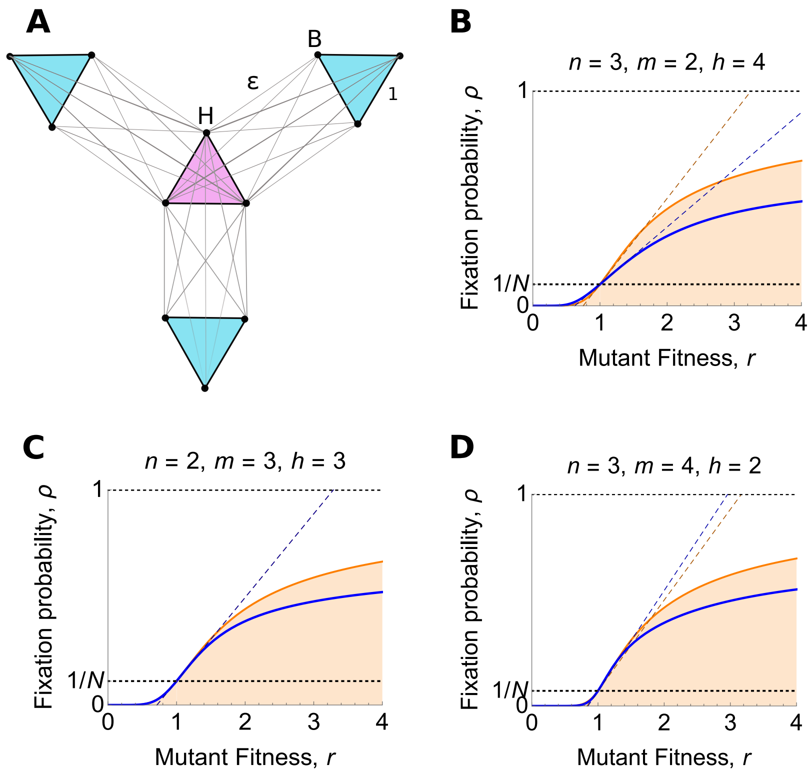

Fig 4. Star of Islands (A) The Star of Islands graph consists of a hub island of size $h \geq 2$, and $n \geq 1$ other islands of size $m \geq 2$. Edge weights are as shown. **(B)–(D)** Blue curves show fixation probability, Eqs. (19)–(21), plotted against mutant fitness r, in the $\epsilon \to 0$ limit. Blue dotted lines show the weak selection result, Eqs. (8) and (18). The orange curve and dotted line show the corresponding well-mixed population results, Eqs. (1) and (4), for comparison. The Star of Islands graph is (B) a suppressor for $m \geq h - 1$, (C) a reducer for $m = h$, and (D) a transient amplifier for $m \geq h + 1$.

We prove in S1 Appendix that the Separated Hubs graph, in the $\epsilon \to 0$ limit, is a suppressor for $n \leq h$, a transient amplifier for $n \geq h + 2$, and a reducer for $n = h + 1$.

Star of Islands

Our final example, the Star of Islands, is similar to Separated Hubs, except that the hubs are connected to each other. It consists of $h \geq 2$ hub vertices and $n \geq 2$ islands, with $m \geq 2$ vertices per island. The total population size is $N = nm + h$. Within the hub and each island, vertices are connected to one another with weight 1. Each hub vertex is also connected to each island vertex with weight $\epsilon > 0$.

For weak selection, in the $\epsilon \to 0$ limit, we calculate

$$N_{\text{eff}} = N + \frac{(m-h)mnh\big(h(h-1) + m(m-1)(n-2)\big)}{\big(h(h-1) + m(m-1)\big)\big(h(h-1) + m(m-1)n\big)}. \tag{18}$$

The second term on the right-hand side has the sign of $m - h$. It follows that the Star of Islands is an amplifier of weak selection when $m > h$, and a suppressor of weak selection when $m < h$.

We show in S1 Appendix that the strongest amplifier of weak selection occurs for $h = 2$, $m = 4$, and first $\epsilon \to 0$ and then $n \to \infty$. In this case $N_{\text{eff}}/N \to 9/7$. The

strongest suppressor occurs for first $\epsilon \to 0$, then $n \to \infty$, and then $h \to \infty$, leading to $N_{\text{eff}}/N \to 0$.

For arbitrary $r > 0$, in the $\epsilon \to 0$ limit, we obtain $\rho_{SI_{n,m,h}(r)} = \text{num}/\text{denom}$ with

$$\begin{aligned} \text{num} = r^m(1-r^{-1})\left(1-r^{-(h+m)}\right) \\ \left(hr^h\left(1-r^{-(h-1)}\right)(mn(m-1)r^m + h(h-1)) \right. \\ \left. + mr^m\left(1-r^{-(m-1)}\right)\left(mn(m-1)+h(h-1)r^h\right)\right), \end{aligned} \tag{19}$$

$$\begin{aligned} \text{denom} = (mn+h)\left(h\left(1-r^{-(h-1)}\right)\right. \\ \left. + mr^m\left(1-r^{-(m-1)}\right)\right)\left(mr^m\left(1-r^{-(m-1)}\right)(1-x^n)\right. \\ \left. + h\left(1-r^{-(h-1)}\right)\left(r^{h+m}-x^n\right)\right), \end{aligned} \tag{20}$$

and

$$x = \frac{mr^{-m}(r^{m-1}-1)+h(r^{h-1}-1)}{mr^h(r^{m-1}-1)+h(r^{h-1}-1)}. \tag{21}$$

In the limit of many islands, this simplifies to

$$\lim_{n\to\infty} \rho_{SI_{n,m,h}}(r) = \begin{cases} 0 & 0 \le r \le 1 \\ \frac{(m-1)(1-r^{-1})\left(1-r^{-(m-1)}\right)}{hr^{-m}\left(1-r^{-(h-1)}\right)+m\left(1-r^{-(m-1)}\right)} & r > 1. \end{cases} \tag{22}$$

We prove in S1 Appendix that the Star of Islands is a reducer for $m = h$.

Approximating fixation probability

We have defined the effective population size N_{eff} in terms of the expected remeeting times of random walks. While this definition allows N_{eff}—and, via Eq. (8), fixation probabilities under weak selection—to be computed in polynomial time, it gives little intuition for how N_{eff} relates to more familiar graph statistics.

To build such intuition, we use a mean-field approximation from Fotouhi et al. [51]. We suppose that each remeeting time τ_i is approximately equal to a single value, τ. Then from Eq. (7) we have

$$1 = \sum_{i\in G} \pi_i^2 \tau_i \approx \tau \sum_{i\in G} \pi_i^2 = \frac{\tau \sum_{i\in G} w_i^2}{(\sum_{i\in G} w_i)^2} = \frac{\tau\mu_2}{N\mu_1^2}.$$

Above, $\mu_1 = \frac{1}{N}\sum_{i\in G} w_i$ and $\mu_2 = \frac{1}{N}\sum_{i\in G} w_i^2$ are the first and second moments, respectively, of the weighted degree distribution. Solving for τ and substituting in the definition of N_{eff} gives the approximation

$$N_{\text{eff}} \approx N\mu_1^2/\mu_2. \tag{23}$$

Substituting in Eq. (8) gives an approximation for fixation probability under weak selection in terms of μ_1 and μ_2. Interestingly, the right-hand side of Eq. (23) was taken as the definition of effective population size by Antal et al. [15], who studied the same model but arrived at this expression by different methods and assumptions.

The approximation in Eq. (23) is reasonably accurate when compared to exact numerical calculation of N_{eff}/N for Erdös-Renyi and Barabási-Albert graphs (Fig. 5).

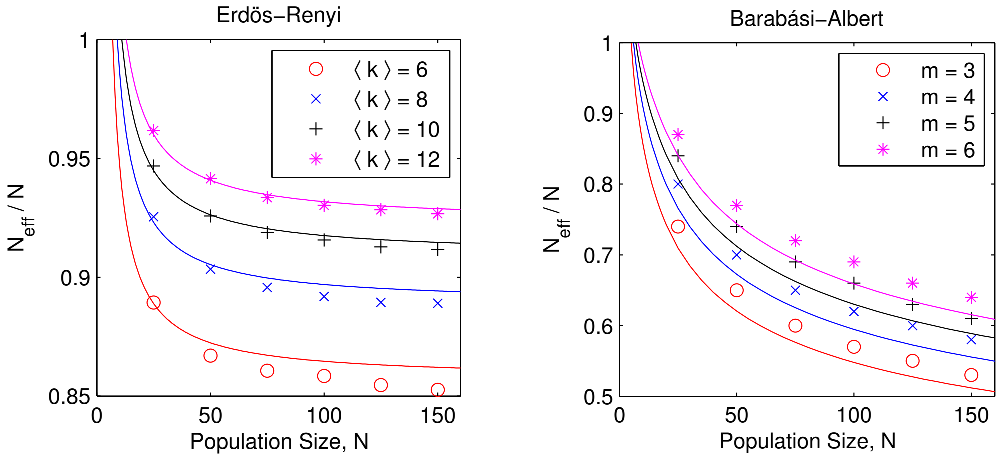

A **B**

Fig 5. Random graphs suppress weak selection. Plot markers show the ratio N_{eff}/N, averaged over 1000 trials, plotted against population size N. Effective population size, N_{eff}, is calculated by numerically solving Eq. (5) for each graph and applying Eqs. (6) and (9). All random graphs generated have $N_{\text{eff}} < N$ and are therefore suppressors of weak selection. Curves of the corresponding colors show the approximation $N_{\text{eff}}/N \approx \mu_1^2/\mu_2$ from Eq. (23). Overall, we find that larger, sparser, and more heterogeneous graphs have smaller N_{eff}/N; these trends are all reflected in the approximation from Eq. (23). **(A)** Erdös-Renyi graphs were generated for specific values of the expected degree $\langle k \rangle$ by setting the link probability to $p = \langle k \rangle/(N-1)$. The moments μ_1 and μ_2 were approximated by assuming that the degree of each vertex is independently distributed as $\text{Binom}(N-1, p)$. This leads to $N_{\text{eff}}/N \approx (N-1)p/[(N-2)p+1]$. At the minimum population size of $N = \langle k \rangle + 1$, the graph is complete and therefore $N_{\text{eff}}/N = 1$. **(B)** Barabási-Albert preferential attachment networks [52] were generated for linking numbers $3 \leq m \leq 6$, starting from a complete graph of size $m+2$. The second moment was calculated using the expected degree distribution for finite Barabási-Albert networks obtained by Fotouhi and Rabbat [53]. At the minimum population size of $N = m+2$, the graph is complete and therefore $N_{\text{eff}}/N = 1$.

In particular, the approximation explains the general trend that larger, sparser, and more heterogeneous graphs act as stronger suppressors (have smaller N_{eff}/N ratio). We note, however, that since $\mu_2 \leq \mu_1^2$ for any degree distribution, the approximated N_{eff} in Eq. (23) is at most equal to the actual population size N, with equality only for isothermal graphs. Therefore, the possibility of amplifiers of weak selection is not seen in this approximation.

Discussion

Weak-selection methodology

We have brought the method of weak selection, previously developed to analyze games on graphs [4, 5, 9–12, 14, 43], to bear on the question of amplifiers and suppressors. While our focus is on death-Birth updating, the method also applies to Birth-death updating, using a modified version of the coalescing random walk [5, 50]. Our weak-selection method has the advantage of being computable in polynomial time (in the size of the graph), in contrast to other numerical methods [22, 25, 26, 32], which take exponential time. Our expression for fixation probabilities in terms of coalescence times, Eq. (8), also enables the proof of general results such as the Weak-Selection Isothermal Theorem for dB. A drawback of the weak-selection approach is that it does not distinguish between transient and non-transient amplifiers, nor can it detect complex behavior such as multiple switchings between amplification and suppression [26].

Effective population size

Our analysis motivated a new definition of the effective population size of a graph, $N_{\text{eff}} = \sum_{i \in G} \pi_i \tau_i$. This notion of effective population is particular to dB updating, since it was derived from weak-selection fixation probabilities under this update rule. Our definition has a number of interesting connections to other definitions previously proposed for this concept [15, 37–39].

First, as noted above, the effective population size of Antal et al. [15] appears in Eq. (23) as an approximation to ours. Whereas we obtain $N_{\text{eff}} \approx N\mu_1^2/\mu_2$ using coalescent theory and assuming uniformity of remeeting times, Antal et al. [15] obtain the same expression using diffusion approximation and assuming degree-uncorrelatedness of the graph. That the same expression arises from distinct analytical frameworks and assumptions hints at its naturality.

Second, our definition differs by a simple rescaling from the notion of "fixation effective population size" proposed by Allen, Dieckmann, and Nowak (hereafter, ADN) [38], and elaborated on by Giaimo et al. [39]:

$$N_{\text{eff}}^{\text{ADN}} = \frac{N^2}{N-1} \frac{d\rho}{dr}\bigg|_{r=1}. \tag{24}$$

Comparing Eqs. (24) and (8), we find the relationship

$$N_{\text{eff}}^{\text{ADN}} = \frac{N(N_{\text{eff}} - 2)}{2(N-1)}.$$

For large populations, $N_{\text{eff}}^{\text{ADN}} \approx N_{\text{eff}}/2$. The factor of two appears because the ADN definition uses the Wright-Fisher (discrete generations) model as a baseline, whereas the baseline for our N_{eff} is the death-Birth process, for which generations are overlapping. Such factors of two commonly appear in translating between discrete- and overlapping-generations models [35, 38, 54].

Third, our proposed definition is closely related to the concept of "inbreeding effective population size", which dates back to Wright [33] and has been elaborated on by many others [34–37]. The inbreeding effective population size is typically defined, for diploid populations, as the size of an idealized population that would have the same level of autozygosity (a locus containing two alleles that are identical by descent) [34, 36]. Although autozygosity as such cannot occur in haploid populations, the remeeting time τ_i quantifies the closely-related concept of *auto-coalescence*—the time for two hypothetical, independent lineages from i to coalesce. For rare mutation, coalescence time is proportional to the probability of non-identity by descent [55]; thus auto-coalescence can be taken as a proxy for autozygosity in haploid populations. Our N_{eff} is equal to the size of a well-mixed population that would experience the same degree of auto-coalescence, when averaged over individuals weighted by their reproductive values π_i. It is therefore reasonable to interpret our N_{eff} as a haploid analogue of the inbreeding effective population size.

Transient amplifiers of selection

The most novel of our results is the discovery of the first transient amplifiers of selection for dB updating. Previous investigations [17, 22, 27] had uncovered only suppressors and reducers. Of the transient amplifiers we have found, the strongest is the 2-Fan, $F_{n,2}$, with many blades ($n \to \infty$; Fig. 2D). A companion work [45] proves that full (non-transient) amplifiers cannot exist for death-Birth updating.

Transient amplifiers appear to be quite rare for death-Birth updating. None were present within an ensemble of thousands of small graphs analyzed by Hindersin and Traulsen [22]. Similarly, no amplifiers of weak selection for dB were found in our ensembles of Erdös-Renyi and Barabasi-Albert random graphs.

Why should transient amplifiers be so rare? One possible clue comes from the approximation for effective population size in Eq. (23). The approximated N_{eff} is always less than or equal to the actual population size N, with equality only for isothermal graphs. Thus any amplifier (transient or not) must be a graph for which the approximation in Eq. (23) is inaccurate. Another possible clue is found by combining Eqs. (9) and (7) to obtain

$$\frac{N - N_{\text{eff}}}{N^2} = \left(\frac{1}{N}\sum_{i \in G} \pi_i^2 \tau_i\right) - \left(\frac{1}{N}\sum_{i \in G} \pi_i\right)\left(\frac{1}{N}\sum_{i \in G} \pi_i \tau_i\right).$$

The right-hand side can be interpreted as the covariance of π_i with $\pi_i\tau_i$, as i runs over vertices of G. It follows that G is an amplifier of weak selection if and only if π_i and $\pi_i\tau_i$ are negatively correlated on G. This requires a very strong negative relationship between weighted degree and remeeting time, which seems unlikely to arise in the usual random graph models. A third clue comes from a companion work [45], which proves a bound on the strength of transient amplifiers for dB. Since transient amplifiers are limited in their possible strength, it is reasonable to suppose they are also limited in number. Each of these clues, however, falls very short of a formal proof.

Reducers of fixation

Evolutionarily speaking, reducers of fixation maintain the status quo. They protect the resident type from replacement by any mutation, whether beneficial or deleterious. Reducers may have applications in bio-engineering, in situations where it is desirable to inhibit the accumulation of all fitness-affecting mutations. Indeed, it has been argued that the cycle-like structure of epithelial stem cells in mammals [56, 57] may have been evolutionarily designed to limit somatic mutations [29]. The cycle was the first known

reducer [22]; others were identified by Hindersin et al. [29]. To these examples we have added two more: the Separated Hubs graph with $n = h + 1$ and the Star of Islands with $m = h$.

Isothermal graphs appear to be obvious candidates for reducers of fixation. This is because, if G is a reducer of fixation, then $\rho_G(r)$ and $\rho_{K_N}(r)$ must coincide to first order in r at $r = 1$, and this latter property holds for all isothermal graphs according to the Weak-Selection Isothermal Theorem for dB. Indeed, all previously-known examples of reducers [22, 29] were isothermal. However, neither the Separated Hubs graph for $n = h + 1$ nor the Star of Islands for $m = h$ are isothermal; thus reducers need not be isothermal. The converse question—whether all isothermal graphs are reducers—remains open. To resolve this question, one would have to either discover or rule out other behaviors for isothermal graphs G, such as $\rho_G(r) > \rho_{K_N}(r)$ for all r sufficiently close but not equal to 1. Another open question is whether reducers of fixation exist for Bd updating.

Limitations

Although we have uncovered an interesting range of behaviors for dB updating on graphs, there are limitations to our approaches. All of our analytical results involve the limit of either weak selection or certain edge weights going to zero. Some of our results combine these limits, meaning that they apply only in rather extreme scenarios, and the results may depend on the limit ordering [58].

We also do not consider the issue of fixation time [32, 40–42, 59–62]. Previous work [41, 42, 59, 60] has uncovered a tradeoff between fixation probability and time: Graphs that amplify selection tend to have larger fixation times than the complete graph, which impedes their ability to accelerate adaptation. A number of our examples involve limits as certain edge weights go to zero. Fixation times diverge to infinity for these examples; therefore they do not hasten the accumulation of beneficial mutations. The search for graphs that (transiently) amplify selection without greatly increasing fixation times is left to future work.

Conclusion

The identification of amplifiers and suppressors of selection has become a robust field of inquiry [3, 7, 8, 15–28, 39, 41]. Most investigations of this question follow the lead of the initial work [3] in focusing on Birth-death updating. This is an interesting contrast to the study of games on graphs [4, 5, 9–14], which typically considers death-Birth updating—likely because Birth-death updating tends not to support cooperative behaviors [4].

Since the choice of update rule has such marked consequences, a full understanding of evolutionary dynamics in structured populations requires studying a variety of update rules. Indeed, the update rule should properly be considered an aspect of the population structure, equal in importance to the graph itself [11, 27, 28, 50]. If the theory of amplifiers and suppressors is to find application (for example, to microbial populations [8]), it is critical to determine which update rules are plausible for specific organisms. Our work shows that dB updating exhibits at least some of the interesting phenomena that have been observed for Bd updating, and suggests there is more to be discovered.

Supporting information

S1 Appendix Mathematical derivations and proofs.

Acknowledgments

This work was supported by the National Science Foundation Award #1715315. We are grateful to Martin A. Nowak, Josef Tkadlec, and Andreas Pavlogiannis for enlightening conversations.

References

1. Nowak MA, May RM. Evolutionary games and spatial chaos. Nature. 1992;359(6398):826–829.

2. Hauert C, Doebeli M. Spatial structure often inhibits the evolution of cooperation in the snowdrift game. Nature. 2004;428(6983):643–646.

3. Lieberman E, Hauert C, Nowak MA. Evolutionary dynamics on graphs. Nature. 2005;433(7023):312–316.

4. Ohtsuki H, Hauert C, Lieberman E, Nowak MA. A simple rule for the evolution of cooperation on graphs and social networks. Nature. 2006;441:502–505.

5. Allen B, Lippner G, Chen YT, Fotouhi B, Momeni N, Yau ST, et al. Evolutionary dynamics on any population structure. Nature. 2017;544(7649):227–230.

6. Allen B, Sample C, Dementieva Y, Medeiros RC, Paoletti C, Nowak MA. The Molecular Clock of Neutral Evolution Can Be Accelerated or Slowed by Asymmetric Spatial Structure. PLOS Computational Biology. 2015;11(2):e1004108. doi:10.1371/journal.pcbi.1004108.

7. Shakarian P, Roos P, Johnson A. A review of evolutionary graph theory with applications to game theory. Biosystems. 2012;107(2):66–80.

8. Pavlogiannis A, Tkadlec J, Chatterjee K, Nowak MA. Construction of arbitrarily strong amplifiers of natural selection using evolutionary graph theory. Communications Biology. 2018;1(1):71.

9. Taylor PD, Day T, Wild G. Evolution of cooperation in a finite homogeneous graph. Nature. 2007;447(7143):469–472.

10. Chen YT. Sharp benefit-to-cost rules for the evolution of cooperation on regular graphs. The Annals of Applied Probability. 2013;23(2):637–664.

11. Débarre F, Hauert C, Doebeli M. Social evolution in structured populations. Nature Communications. 2014;5:4409.

12. Durrett R. Spatial evolutionary games with small selection coefficients. Electronic Journal of Probability. 2014;19(121):1–64. doi:10.1214/EJP.v19-3621.

13. Peña J, Wu B, Arranz J, Traulsen A. Evolutionary games of multiplayer cooperation on graphs. PLOS Computational Biology. 2016;12(8):e1005059.

14. Fotouhi B, Momeni N, Allen B, Nowak MA. Conjoining uncooperative societies facilitates evolution of cooperation. Nature Human Behaviour. 2018;2:492–499.

15. Antal T, Redner S, Sood V. Evolutionary dynamics on degree-heterogeneous graphs. Physical Review Letters. 2006;96(18):188104.

16. Broom M, Rychtář J. An analysis of the fixation probability of a mutant on special classes of non-directed graphs. Proceedings of the Royal Society A: Mathematical, Physical and Engineering Science. 2008;464(2098):2609–2627.

17. Frean M, Baxter G. Death-birth ordering and suppression of fitness in networks; 2008. Available from: `http://homepages.mcs.vuw.ac.nz/marcus/manuscripts/FreanBaxterJTB.pdf`.

18. Voorhees B, Murray A. Fixation probabilities for simple digraphs. Proceedings of the Royal Society A: Mathematical, Physical and Engineering Science. 2013;469(2154):20120676.

19. Monk T, Green P, Paulin M. Martingales and fixation probabilities of evolutionary graphs. Proceedings of the Royal Society A: Mathematical, Physical and Engineering Science. 2014;470(2165):20130730.

20. Adlam B, Chatterjee K, Nowak M; The Royal Society. Amplifiers of selection. Proceedings of the Royal Society A: Mathematical, Physical and Engineering Science. 2015;471(2181):20150114.

21. Cuesta FA, Sequeiros PG, Rojo ÁL. Fast and asymptotic computation of the fixation probability for Moran processes on graphs. Biosystems. 2015;129:25–35.

22. Hindersin L, Traulsen A. Most undirected random graphs are amplifiers of selection for birth-death dynamics, but suppressors of selection for death-birth dynamics. PLOS Computational Biology. 2015;11(11):e1004437.

23. Jamieson-Lane A, Hauert C. Fixation probabilities on superstars, revisited and revised. Journal of Theoretical Biology. 2015;382:44–56.

24. Pavlogiannis A, Tkadlec J, Chatterjee K, Nowak MA. Amplification on undirected population structures: comets beat stars. Scientific Reports. 2017;7(1):82.

25. Cuesta FA, Sequeiros PG, Rojo ÁL. Suppressors of selection. PLOS One. 2017;12(7):e0180549.

26. Cuesta FA, Sequeiros PG, Rojo ÁL. Evolutionary regime transitions in structured populations. PLOS One. 2018;13(11):e0200670.

27. Kaveh K, Komarova NL, Kohandel M. The duality of spatial death–birth and birth–death processes and limitations of the isothermal theorem. Royal Society Open Science. 2015;2(4):140465.

28. Pattni K, Broom M, Rychtář J, Silvers LJ; The Royal Society. Evolutionary graph theory revisited: when is an evolutionary process equivalent to the Moran process? Proceedings of the Royal Society A: Mathematical, Physical and Engineering Science. 2015;471(2182):20150334.

29. Hindersin L, Werner B, Dingli D, Traulsen A. Should tissue structure suppress or amplify selection to minimize cancer risk? Biology Direct. 2016;11(1):41.

30. Cox JT. Coalescing random walks and voter model consensus times on the torus in $\mathbb{Z}^d$. Annals of Probability. 1989;17(4):1333–1366.

31. Liggett TM. Interacting Particle Systems. Springer Science & Business Media; 2006.

32. Hindersin L, Möller M, Traulsen A, Bauer B. Exact numerical calculation of fixation probability and time on graphs. Biosystems. 2016;150:87–91.

33. Wright S. Evolution in Mendelian populations. Genetics. 1931;16(2):97.

34. Kimura M, Crow JF. The measurement of effective population number. Evolution. 1963;17(3):279–288.

35. Felsenstein J. Inbreeding and variance effective numbers in populations with overlapping generations. Genetics. 1971;68(4):581.

36. Crow JF, Denniston C. Inbreeding and variance effective population numbers. Evolution. 1988;42(3):482–495.

37. Broom M, Voelkl B. Two measures of effective population size for graphs. Evolution: International Journal of Organic Evolution. 2012;66(5):1613–1623.

38. Allen B, Nowak MA, Dieckmann U. Adaptive dynamics with interaction structure. The American Naturalist. 2013;181(6):E139–E163.

39. Giaimo S, Arranz J, Traulsen A. Invasion and effective size of graph-structured populations. PLOS Computational Biology. 2018;14(11):e1006559.

40. Hathcock D, Strogatz SH. Fitness dependence of the fixation-time distribution for evolutionary dynamics on graphs. arXiv preprint arXiv:181205652. 2018;.

41. Möller M, Hindersin L, Traulsen A. Exploring and mapping the universe of evolutionary graphs identifies structural properties affecting fixation probability and time. Communications Biology. 2019;2(1):137.

42. Tkadlec J, Pavlogiannis A, Chatterjee K, Nowak MA. Population structure determines the tradeoff between fixation probability and fixation time. Communications Biology. 2019;2.

43. Allen B, Nowak MA. Games on graphs. EMS Surveys in Mathematical Sciences. 2014;1(1):113–151.

44. Hadjichrysanthou C, Broom M, Rychtář J. Evolutionary games on star graphs under various updating rules. Dynamic Games and Applications. 2011;1(3):386.

45. Tkadlec J, Pavlogiannis A, Chatterjee K, Nowak MA. Limits on amplifiers of natural selection under death-Birth updating;.

46. Haldane JBS. A Mathematical Theory of Natural and Artificial Selection I. Transactions of the Cambridge Philosophical Society. 1924;23:19–41.

47. Kimura M. On the probability of fixation of mutant genes in a population. Genetics. 1962;47(6):713.

48. Akashi H, Osada N, Ohta T. Weak selection and protein evolution. Genetics. 2012;192(1):15–31.

49. Nowak MA, Sasaki A, Taylor C, Fudenberg D. Emergence of cooperation and evolutionary stability in finite populations. Nature. 2004;428(6983):646–650.

50. Allen B, McAvoy A. A mathematical formalism for natural selection with arbitrary spatial and genetic structure. Journal of Mathematical Biology. 2018;doi:10.1007/s00285-018-1305-z.

51. Fotouhi B, Momeni N, Allen B, Nowak MA. Evolution of cooperation on large networks with community structure. Journal of the Royal Society Interface. 2019;16(152):20180677.

52. Barabási AL, Albert R. Emergence of scaling in random networks. Science. 1999;286(5439):509–512.

53. Fotouhi B, Rabbat MG. Network growth with arbitrary initial conditions: Degree dynamics for uniform and preferential attachment. Physical Review E. 2013;88(6):062801.

54. Moran PAP. The Statistical Process of Evolutionary Theory. Clarendon Press; 1962.

55. Slatkin M. Inbreeding coefficients and coalescence times. Genetical Research. 1991;58(02):167–175.

56. Bozic I, Nowak MA. Unwanted evolution. Science. 2013;342(6161):938–939.

57. Vermeulen L, Morrissey E, van der Heijden M, Nicholson AM, Sottoriva A, Buczacki S, et al. Defining stem cell dynamics in models of intestinal tumor initiation. Science. 2013;342(6161):995–998.

58. Sample C, Allen B. The limits of weak selection and large population size in evolutionary game theory. Journal of Mathematical Biology. 2017;75(5):1285–1317.

59. Frean M, Rainey PB, Traulsen A. The effect of population structure on the rate of evolution. Proceedings of the Royal Society B: Biological Sciences. 2013;280(1762):20130211.

60. Hindersin L, Traulsen A. Counterintuitive properties of the fixation time in network-structured populations. Journal of The Royal Society Interface. 2014;11(99):20140606.

61. Askari M, Samani KA. Analytical calculation of average fixation time in evolutionary graphs. Physical Review E. 2015;92(4):042707.

62. Farhang-Sardroodi S, Darooneh AH, Nikbakht M, Komarova NL, Kohandel M. The effect of spatial randomness on the average fixation time of mutants. PLOS Computational Biology. 2017;13(11):1–22. doi:10.1371/journal.pcbi.1005864.

Figure 1

Click here to download Figure WMcyclestar.pdf

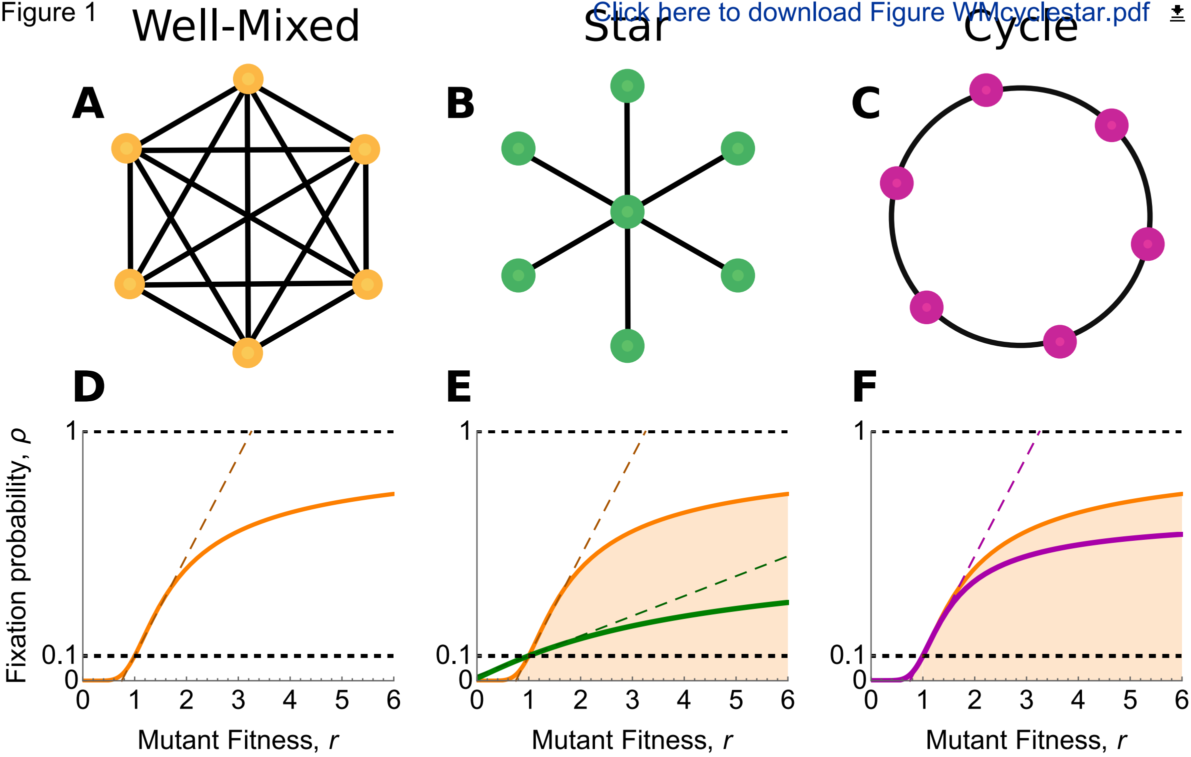

Figure 2
Click here to download Figure FamCombined.pdf

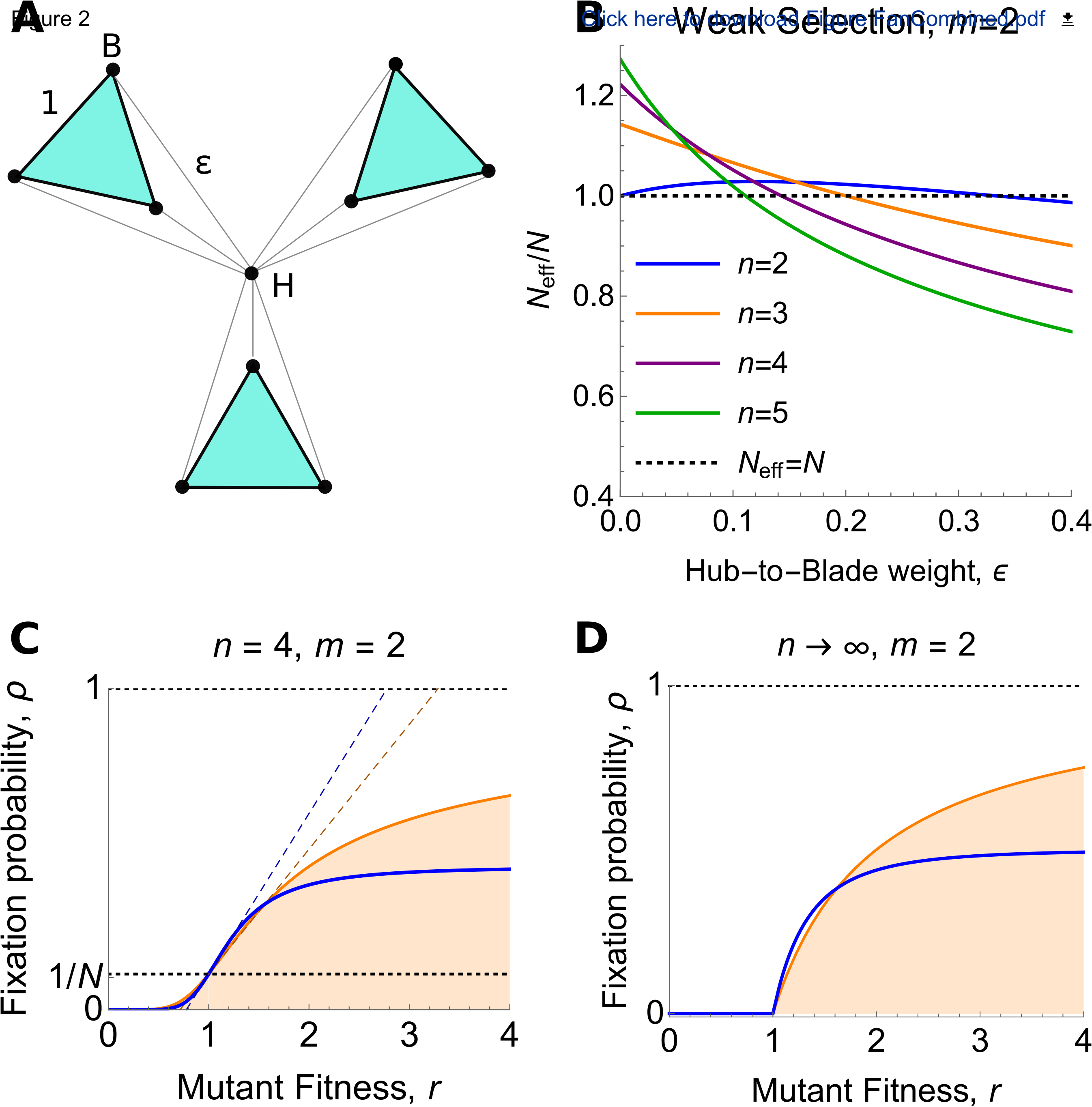

Figure 3

Click here to download Figure SeparatedHubsCombined.pdf

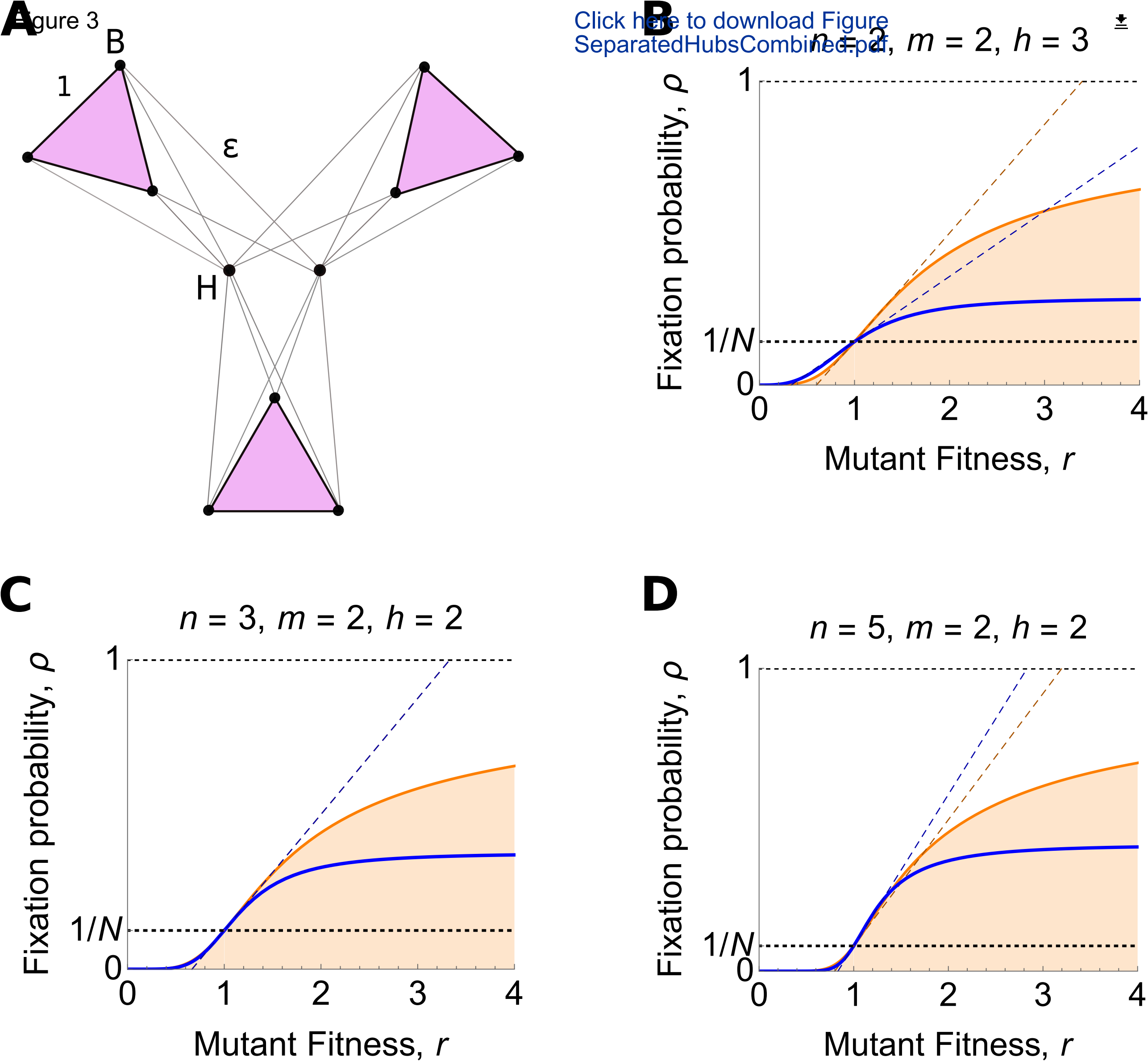

Figure 4

Click here to download Figure StarIslandsCombined.pdf

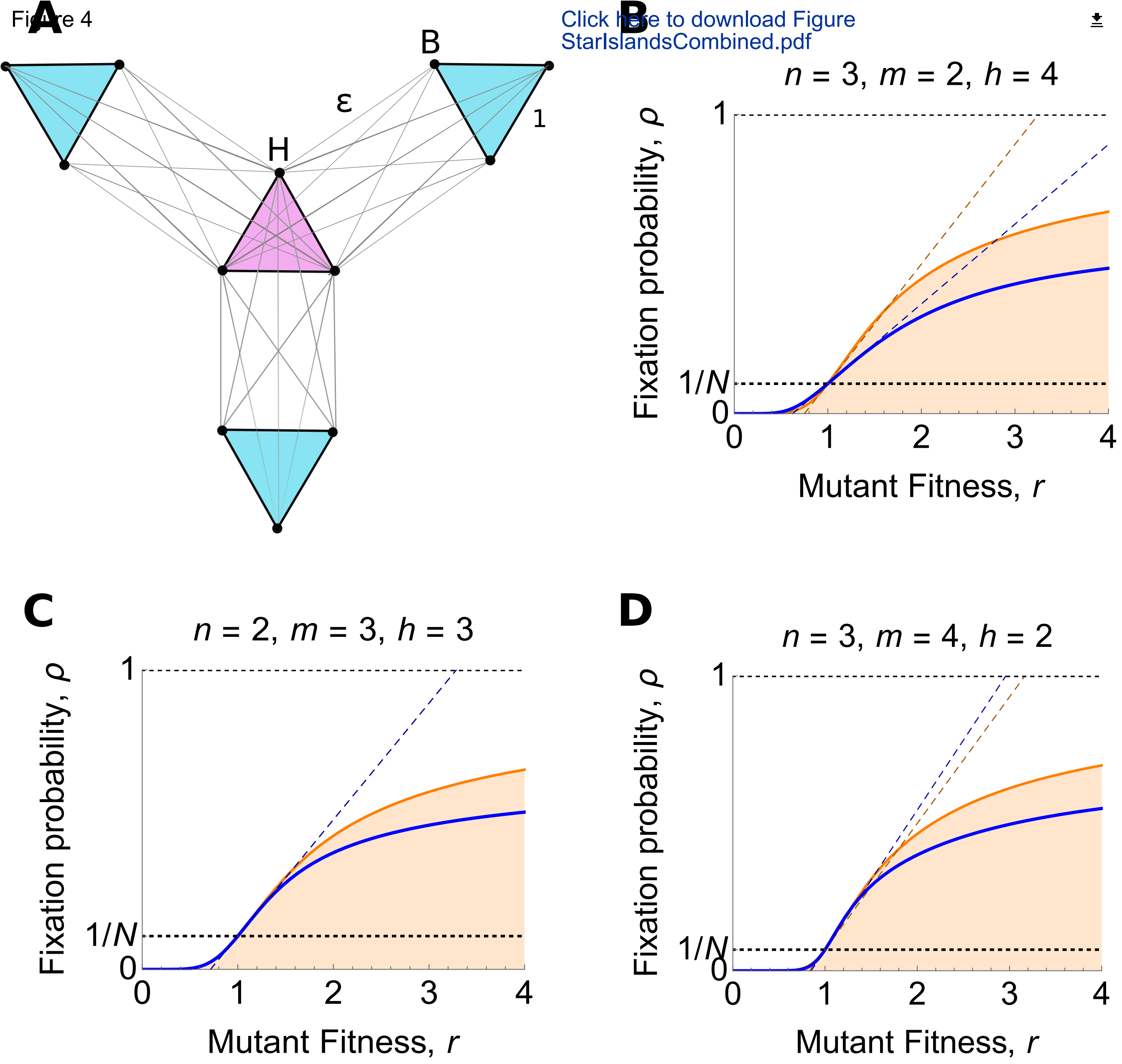

Figure 5A

Click here to download Figure ER-

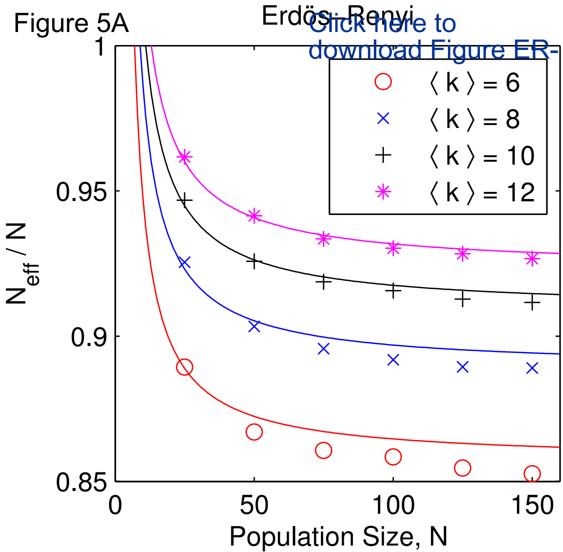

Figure 5B

Click here to download Figure BA-

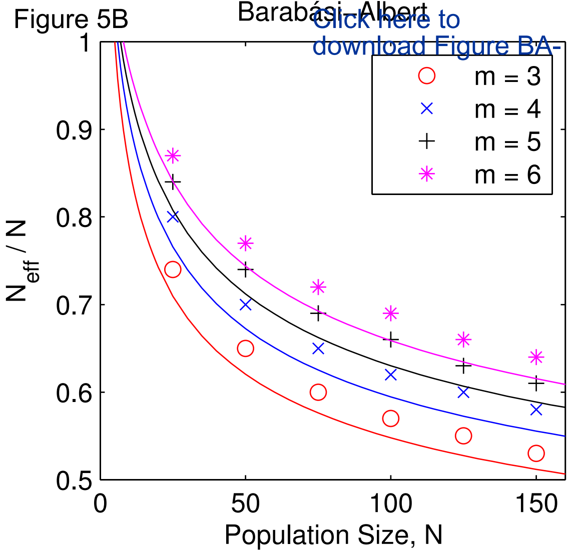

Click here to access/download
Supporting Information
SI.pdf
