# Supplementary material for: Transient amplifiers of selection and reducers of fixation for death-Birth updating on graphs
Source: PLoS Comput Biol. 2020 Jan 17;16(1):e1007529. doi: 10.1371/journal.pcbi.1007529 (PMC6968840; doi:10.1371/journal.pcbi.1007529)
Supplement: Attachment — Submitted filename: Transient_Comments.pdf [file pcbi.1007529.s005.pdf]

1 General Comments

Overall, I consider the paper to be very interesting and well reasoned. Especially the way the concept of effective population size is defined and used seems like a very useful new addition to the research in general, and the example of a fan seems to be a graph not considered before with unusual properties.
Unfortunately, the supplementary has a number of minor mistakes that I'd like to be changed.

2 Comments on the Main Manuscript

I don't really have any major complaints about the main manuscript, so I'm just going to give a few points that I thought were worth considering. But as far as I have seen, nothing really needs to be changed.

1. I'd like to mention that the situation of amplifiers under dB is similar to the situation of suppressors under Bd. In general, there seems to be a rather low number of suppressors under Bd, and most of them seem to be transient and/or weak, at least for unweighted and undirected graphs. The fan also seems very interesting for Bd. A quick estimate left me with the conclusion that it is probably an unusually strong suppressor under Bd, in essence only fixating if the mutant lands in the central node as long as $r << 1/\epsilon$ and $n << 1/\epsilon$ if I didn't make a mistake.

2. Could it be possible to use the effective population size to prove that there is no undirected, unweighted graph which is an amplifier under dB (at least in weak selection) by showing that $N_{eff} > N$ requires weights or directedness? If not, what big classes of graphs can be proven to have $N_{eff} < N$?

3. Likewise, what is the relationship between the coalescence/remeeting time and fixation time? It seems intuitive that there should be a connection, and the paper shows how the remeeting time relates to fixation probability, at least in weak selection. Since there seems to be a broad correlation between in fixation probability and time (positive in Bd, negative in dB), this could be a good angle to explain this correlation.

3 Comments on the Supplementary Information

As already mentioned, there are a few oversights in the supplementary that I'd like to be remedied.

1. The numbering of the equations is wrong on almost every reference. It seems like one or two equations were removed without changing the references, since it is usually just off by one or two.

2. On page 5, equation 12, I do not quite understand how the x_j relates to the $x_i^{(2)}$ in equation 10. And as a result I do not understand how equation 13 follows from 10 and 12 (maybe the other equation clears this up, which unfortunately seems to have been removed, since it's just written as (??)). I would like this part to be explained a bit more.

3. On page 8, the derivations of equations 33-36 are not explained very well. Especially, when I tried to replicate them, I concluded that $\tau_{HH'} = 1 + nmp_{HB}\tau_{HB}$ which is slightly but importantly different from the shown equation 33, and similar differences for the rest of the equations. Maybe I did a mistake, but if so, I'd like it to be explained to me. Otherwise, this needs to be changed.

4. I found some further small mistakes: On page 8, you wrote sometimes w_h instead of w_H. On page 12 in the first part of the text before the first equation, if I'm not mistaken, it needs to be $b = nm + n$, not $b = nm + m$. There is also a [?] on page 14. It would be nice if not only these were fixed, but also if the authors would read carefully through the paper again whether there are more of these mistakes.
