# Supplementary material for: Transient amplifiers of selection and reducers of fixation for death-Birth updating on graphs
Source: PLoS Comput Biol. 2020 Jan 17;16(1):e1007529. doi: 10.1371/journal.pcbi.1007529 (PMC6968840; doi:10.1371/journal.pcbi.1007529)
Supplement: Attachment — Submitted filename: dB graphs response to reviewers.pdf [file pcbi.1007529.s006.pdf]

We thank the reviewers for all of their helpful comments, especially for catching some of our typos and other errors. All errors have been resolved, and we have checked the manuscript and SI thoroughly to make sure there are none remaining.

In addition, we have added new material in response to the reviewer feedback:

- We have added a new section on perturbing isothermal graphs to create amplifiers of weak selection. Using this method, we were able to identify an unweighted amplifier of weak selection, which resolves a question of Reviewer #3.
- In response to a request from Reviewer #2, we have performed Monte Carlo simulations to verify our mathematical methods. As expected, there is excellent agreement between the two. These simulations are presented in the SI.

Reviewer #1: A very well written paper that can be accepted as is.

Only one minor issue: There is a missing reference to an equation in the supplemental material (on the line between eqs (12) and (13))

Thank you--this has been fixed

Reviewer #2: This submission is nice, with interesting results that are relatively easy to read, and good tie-ins to existing literature.

My only substantial problem with the submission is in the sections evaluating the methodology for particular graphs, specifically a 'fan.' I see two problems.

First, the analysis investigates a limit of the fixation probability as epsilon approaches 0. But in that limit, the graphs are no longer well-behaved in the sense that their fixation time goes to infinity. In other words, the graph cannot achieve fixation in finite time in the limit that the blades are not connected to the hub. So this limit does not make sense to me, because the graph reduces to independent Moran processes, one on each blade.

We understand the confusion here, as it is true that the fixation probability is undefined at $\epsilon=0$. However, the limit is well-defined in the usual mathematical sense that the fixation probability can be made arbitrarily close to its limiting value by choosing ϵ sufficiently small. In other words, the limiting value is an accurate approximation for small but positive ϵ. This point is corroborated by our new Figure S1, in which we show excellent agreement between the $\epsilon \to 0$ limit and Monte Carlo simulations for $\epsilon = 0.001$.

Since this confusion is likely to be shared with other readers, we have added some explanation about the $\epsilon \to 0$ limit on lines 222-225.

Second, there is an error in computing that limit for the fan from Eq. 13. Lim eps -> 0 of Neff (Eq. 13) is mn. Either the equation is misreported or there is a deeper issue to be addressed.

We thank you very much for identifying this mistake. Eq. (13) was misreported--we had simply pasted in the wrong result. We have replaced this equation with its correct version (now Eq. (14)), which resolves the issues you mentioned. The new Eq. (14) result has the correct limits, agrees with the corresponding result for Separated Hubs in the SI, and also agrees with the results for nonweak selection.

I would also be interested to see a comparison of the theoretical results with simulations that approximate fixation over a large number of trials. For example, in Fig 2 panels C and D, I suggest the authors present simulation results of fixation probability as dots for various values of r and show that Eq 14 passes through them when r is approximately 1. Same thing for Figs 3 and 4. Showing agreement between theory and simulations justifies your methodology and assures the reader that your math is right.

We thank you for the suggestion to run Monte Carlo simulations. We have run simulations on all three graph structures, in two cases: (i) for $\epsilon=0.1$, to verify our weak-selection results, and (ii) for $\epsilon = 0.001$, to verify our $\epsilon \to 0$ results. Both sets of simulations show excellent agreement with our analytical results. As expected, the simulated fixation probability coincides with the weak-selection tangent line around r=1, but deviates as r moves away from 1.

We present the Monte Carlo results in Supplementary Figures 1 and 2.

There are a number of additional minor revisions that I include in the attached, including suggested references, interpretations, a request for an additional clarifying figure, etc.

We thank you for all of the corrections and suggestions in the PDF comments. We have accepted all of them, with the following comments:

- With regard to the initial placement of the mutant, we clarify on lines 83-86 that we are considering the initial mutant location to be uniformly distributed over all vertices. This is appropriate for death-Birth updating, since each vertex is replaced with uniform probability at every time-step.
- While there is probably some connection between Eq. (7) and Wald's identity (and we thank you for suggesting this), the more direct connection is to Kac's formula for mean return times. We have added a reference to Kac's formula after Eq. (7).
- A fan with just one blade, or with one vertex per blade, has very different properties that are not characteristic of the fan structure overall. (Specifically, a fan with one vertex per blade is simply a star, which behaves very differently from a fan.) Some of our formulas do not apply in these cases. Rather than introducing special cases for n=1 and m=1, we found it simpler and clearer to restrict to n>=2 and m>=2.

- For these figures (now 3CD, 4BCD, and 5BCD), we did not use a numerical value of ϵ, but rather we used the analytically-calculated limits as $\epsilon \to 0$. This is specified in the figure captions. These figures are now complemented by the Monte Carlo simulations in Supplementary Figure 2, which show excellent agreement between $\epsilon \to 0$ limits and simulated fixation probabilities for $\epsilon = 0.0001$.

There are unlabelled equations and references in the SI as well.

Thank you for pointing this out. We have gone through the SI carefully to make sure all equations and references are correctly labeled.

Reviewer #3:

1 General Comments

Overall, I consider the paper to be very interesting and well reasoned. Especially the way the concept of effective population size is defined and used seems like a very useful new addition to the research in general, and the example of a fan seems to be a graph not considered before with unusual properties. Unfortunately, the supplementary has a number of minor mistakes that I'd like to be changed.

2 Comments on the Main Manuscript

I don't really have any major complaints about the main manuscript, so I'm just going to give a few points that I thought were worth considering. But as far as I have seen, nothing really needs to be changed.

1. I'd like to mention that the situation of amplifiers under dB is similar to the situation of suppressors under Bd. In general, there seems to be a rather low number of suppressors under Bd, and most of them seem to be transient and/or weak, at least for unweighted and undirected graphs. The fan also seems very interesting for Bd. A quick estimate left me with the conclusion that it is probably an unusually strong suppressor under Bd, in essence only fixating if the mutant lands in the central node as long as r << 1= and n << 1= if I didn't make a mistake.

We thank you for this suggestion. We are currently working on a follow-up manuscript that will apply this same methodology to Birth-death updating. Interestingly, the Fan with Bd updating displays several different regimes of behavior, depending on the value of ϵ.

2. Could it be possible to use the effective population size to prove that there is no undirected, unweighted graph which is an amplifier under dB

(at least in weak selection) by showing that Neff > N requires weights or directedness? If not, what big classes of graphs can be proven to have Neff < N?

We have taken up your suggestion to search for unweighted amplifiers of weak selection for death-Birth. We have found that such graphs do exist--the smallest we found is size 12. Our search was informed by a perturbation argument, which suggests that amplifiers of weak selection can be found by starting with an isothermal graph and then reducing the edge weight to vertices with large remeeting time.

We present our newly-discovered unweighted amplifier in Figure 2. The perturbation argument referenced above is now included under main-text results, lines 194-208, with derivation in Section 3 of the S1 Appendix.

3. Likewise, what is the relationship between the coalescence/remeeting time and fixation time? It seems intuitive that there should be a connection, and the paper shows how the remeeting time relates to fixation probability, at least in weak selection. Since there seems to be a broad correlation between in fixation probability and time (positive in Bd, negative in dB), this could be a good angle to explain this correlation.

We agree with you that there is likely a connection between effective population size and fixation time. Our preliminary investigations suggest that this is a deep and interesting question, which probably merits its own paper.

3 Comments on the Supplementary Information

As already mentioned, there are a few oversights in the supplementary that I'd like to be remedied.
1. The numbering of the equations is wrong on almost every reference. It seems like one or two equations were removed without changing the references, since it is usually just off by one or two.

We thank you for pointing out these oversights. We have fixed the equation numbering.

2. On page 5, equation 12, I do not quite understand how the xj relates to the x_i^{(2)} in equation 10. And as a result I do not understand how equation 13 follows from 10 and 12 (maybe the other equation clears this up, which unfortunately seems to have been removed, since it's just written as (??)). I would like this part to be explained a bit more.

We have added intermediate steps so that the derivation is more clear--see Eqs. (12)-(15) of the revised S1 Appendix.

3. On page 8, the derivations of equations 33-36 are not explained very well. Especially, when I tried to replicate them, I concluded that HH0 = 1 + nmpHBHB which is slightly but importantly different from the shown equation 33, and similar differences for the rest of the equations. Maybe I did a mistake, but if so, I'd like it to be explained to me. Otherwise, this needs to be changed.

You are correct in your derivation. Fortunately, we had used the correct equations in our derivations--the mistakes only occurred when transcribing these equations into the SI.

We fixed the typos in Eqs. (33), (34) and (36) (the system of equations is now labeled Eqs. (36) - (39)) and added explanations of their derivations. We also added similar explanations for the derivation of the τ_{ij}'s for the Star of Islands graph (now labeled Eqs. (70) - (73)).

4. I found some further small mistakes: On page 8, you wrote sometimes wh instead of wH. On page 12 in the first part of the text before the first equation, if I'm not mistaken, it needs to be b = nm+n, not b = nm+m. There is also a [?] on page 14. It would be nice if not only these were fixed, but also if the authors would read carefully through the paper again whether there are more of these mistakes.

We thank you for pointing out these oversights. We read through the SI again and fixed mistakes, including:

- Changed the instances of wh to wH
- Fixed the ordering of the subscripts on *SH* in Theorem 3 to *n*,*m*,*h*
- Changed b = nm+n from b = nm+m as noted by reviewer
- In the first paragraph of subcase 3.2 of Theorem 3 on page 13, changed subscript from ρK_{nm+n-1} to ρK_{nm+h}
- Fixed exponent in numerator of Eq. (86) (now labeled Eq. (93)) from -(*m* - 1) to -(*m* + *h*)
